# Genome-wide identification of oat *TCP* gene family and expression patterns under abiotic stress

**DOI:** 10.3389/fgene.2025.1533562

**Published:** 2025-02-04

**Authors:** Jiaming Nie, Hongbin Zhao, Xiaodong Guo, Tao Zhang, Bing Han, Huiyan Liu

**Affiliations:** ^1^ Inner Mongolia Agricultural University, Hohhot, China; ^2^ Key Laboratory of Wheat Germplasm Innovation and Utilization Autonomous Region Higher School, Hohhot, China; ^3^ Key Laboratory of Grassland Resources of the Ministry of Education, Hohhot, China

**Keywords:** oat, *TCP* gene family, genome-wide identification, abiotic stress, qRT-PCR

## Abstract

TCP transcription factors are a unique class of transcription factors that play important roles in alleviating abiotic stresses such as drought and salt. In this study, the whole-genome data of three cultivated varieties, namely, “SFS”, “Sang” and “OT3098v2”, were utilized to identify and analyze the members of the *TCP* gene family in oats, and their responses to two abiotic stresses, drought and salt, were also investigated. Results showed that there are 83, 65, and 30 non-redundant *TCP* genes in the three oats, with the highest number of *TCP* genes specific to the “SFS”, reaching 22 genes. The oat *TCP* genes can be classified into three subfamilies: PCF, CIN, and CYC/TB1. Most *AsTCP* genes have important motifs, Motif 1 and Motif 2, which are part of the bHLH domain. Additionally, various cis-acting elements related to hormone response, abiotic stress, light response, and growth and development were found in the promoters of *AsTCP* genes. The main amplification mechanism of the oat *TCP* gene family is fragment duplication. Two tandem duplications, *AsTCP058*/*AsTCP059* and *AsTCP023*/*AsTCP025*, are stably present in the three oats. The highest number of *AsTCP* collinear relationships exist in the “SFS” with 89 pairs. After drought and salt stress treatments, significant differences in gene expression were observed among different oat cultivars and treatment periods. Genes that showed significant expression changes under both treatments (*AsTCP021*, *AsTCP033*, *AsTCP044*, *AsTCP053*, and *AsTCP058*) may play important roles in oat’s response to abiotic stresses. Notably, *AsTCP053* gene was significantly upregulated at 24 h of stress treatment and showed a more sensitive response to salt stress. This study provides insights into the functional characterization of the oat *TCP* gene family and its molecular mechanisms underlying stress tolerance.

## 1 Introduction

TCP (Teosinte branched1/Cycloidea/proliferating cell factor) transcription factors are a class of plant-specific proteins believed to have originated originated from algae and bryophytes. These factors were first identified in maize (TB1), cynoglossum (CYC), and rice (PCF1, PCF2) and named after their initials (Braun et al., 2012). Notably, *TB1* inhibits the growth and development of lateral branches in maize, while loss of its function promotes lateral branch differentiation (Doebley et al., 1997). In contrast, *CYC* is involved in the abortion of dorsal stamens and regulates floral symmetry formation in cynoglossum (Luo et al., 1996). *PCF* is responsible for maintaining chromosome structure and regulating cell cycle progression (Palatnik et al., 2003). These genes share TCP structural domains in their gene structures (Cubas et al., 1999; Kosugi and Ohashi, 1997). The TCP domain is a 59-amino-acid helix-loop-helix (bHLH) structure capable of binding to DNA or facilitating protein-protein interactions (Dhaka et al., 2017; Manassero et al., 2013). Although all *TCP* genes possess the TCP structural domain, its structure varies among different family members. Based on these differences and their evolutionary relationships, *TCP* gene familiars can be categorized into two subfamilies: Class I and Class II (Martín-Trillo and Cubas, 2010).

With the identification and characterization of more *TCP* genes in plants, their functions have been gradually elucidated. These genes play critical roles in regulating plant growth and development. For instance, in *A. thaliana*, *AtTCP14* and *AtTCP15* regulate embryo growth during seed germination through the gibberellin signaling pathway (Resentini et al., 2014),while also influencing leaf cell development and internode elongation (Kieffer et al., 2011). In rice, the overexpression of *OsPCF7* promotes stem height, root length, and tiller number in transgenic seedlings, while increasing the number of panicles and the proportion of filled grains per plant (Li et al., 2020). Additionally, the cucumber *TCP* gene *CsBRC1* effectively controls lateral shoot growth by repressing the expression of *CsPIN3*(Junjun et al., 2019). Beyond their role in plant development, *TCP* genes also play a pivotal role in the adaptation of plants to environmental stresses. Studies have shown that *TCP* genes enhance stress tolerance through various mechanisms, including the regulation of cellular osmotic pressure (Almeida et al., 2017), signal transduction (Guan et al., 2017; Liu et al., 2020), hormone sensitivity (Ding et al., 2019), and the reduction of reactive oxygen species (ROS) accumulation (Mukhopadhyay and Tyagi, 2015). For instance, the overexpression of *PeTCP10* in *A. thaliana* significantly enhances catalase (CAT) activity, which boosts the plant’s antioxidant capacity and improves its salt tolerance during the nutrient growth period (Xu et al., 2021). However, not all *TCP* genes contribute positively to stress tolerance. For example, *ZmTCP14* in maize promotes the accumulation of ROS, which reduces drought tolerance under drought stress conditions (Jiao et al., 2023). In birch, *BpTCP20* enhances salt and drought tolerance by regulating the expression of *BpMYB8* and *BpIAA5*, which reduces the content of ROS and malondialdehyde (MDA) (Li et al., 2024). In wheat, *TaTCP21-A* negatively regulates cold tolerance by repressing the expression of the cold-responsive gene *TaDREB1C* (Kankan et al., 2024). Moreover, in upland cotton, *GbTCP5* directly activates the expression of *GbERD7*, *GbUBC19*, and *GbGOLS2*, thereby significantly enhancing the plant’s ability to adapt to drought and salt stress (Wang et al., 2023).

Oat, a grain-feeding cash crop (Ju et al., 2022), possesses highly productive and high-quality seeds (Gutierrez-Gonzalez et al., 2013). It is characterized by soft and juicy stems and leaves, and is rich in nutrients (Rasane et al., 2015). Oats have shown remarkable adaptability to various geoclimatic regions and adverse environmental conditions, exhibiting higher resilience compared to other feed crops such as rice and wheat. Additionally, oats can serve as pioneer crops for soil improvement (Han et al., 2014). Among the Oat*s* cultivated today, the most prevalent type is the heterozygous hexaploid species (2n = 6x = 42, AADDCC) known as common Oat (*Avena sativa*). The common Oat genome is large and complex, and remains one of the least explored genomes and transcriptomes among cereal crops (Rasane et al., 2015). The publication of the oat genome sequences has opened up new possibilities for analyzing gene families on a genome-wide scale. However, there are still numerous gaps in our understanding of the *TCP* gene family in oats based on the entire gene sequence. Therefore, this study aims to perform a comprehensive analysis of *TCP* genes identified from different oat genomes and elucidate their response mechanisms to salt stress. The findings of this study will provide novel data and insights for a comprehensive understanding of the molecular mechanism of the oat *TCP* gene family’s response to adversity stress.

## 2 Materials and methods

### 2.1 Experimental materials and stress treatments

Naked oat Nei Avena 6 (NY6) and hulled oat Qing Yin 1 (QY1), provided by the Han Bing Oat Breeding Team at Inner Mongolia Agricultural University, were chosen as the primary experimental materials for this study. Uniformly shaped and sized oat seeds were surface-sterilized by immersing them in 2% NaClO for 5 min, rinsed with sterile water, and placed on filter paper soaked in sterile water. The seeds were then dark-incubated at 16°C in a temperature-controlled incubator until 5 cm shoots and primary roots developed. The seedlings were subsequently transferred to a 96-well hydroponic incubator containing Hoagland’s culture medium and grown for 20 days under day/night temperatures of 22/16°C and a photoperiod of 16/8 h. On the 21st day, seedlings were treated with 20% polyethylene glycol (PEG) 6,000 and 100 mM NaCl, while Hoagland’s solution served as the control. Samples from the treatment groups and control group were collected at 0, 2, 4, 8, 12, and 24 h. After sampling, the tissues were rapidly frozen in liquid nitrogen and stored at −80°C. Each sample included three independent biological replicates and technical replicates for qRT-PCR analysis.

### 2.2 Screening and characterization of AsTCP gene family

Genomic data of three oat varieties “SFS”, “Sang” and “OT3098v2” used to identify the oat TCP gene family were obtained from the OatBioDB Biology database (http://waoOat.cn/). Among them, “Sang” and “OT3098v2” are skin oats and “SFS” is a naked oat. Genome files and genome annotation files for rice, maize, wheat, *A. thaliana*, *Brachypodium distachyon*, *A. tauschii*, were obtained from the Ensembl Plants database (http://plants.ensembl.org/index.html). The HMM file for the TCP structural domain (PF03634) was derived from the Pfam database (https://pfam.xfam.org), and generated by training and constructing a large number of sequences known to be in this gene family. Using the built-in algorithm of HMMER 3.0 software, the input gene sequences were compared with the HMM model to generate the Score value for match strength and the E-value for statistical significance. According to the screening criteria, genes with E-value below 0.01 were initially labelled as candidate genes (Finn et al., 2011). To improve the identification accuracy, all genes identified by HMMER 3.0 (including a few genes with E-value greater than 0.01) were submitted to the NCBI CDD database (https://www.ncbi.nlm.nih.gov/cdd/) for structural domain validation (Lu et al., 2020). Eventually, genes with TCP structural domains were confirmed as members of the *TCP* gene family. *TCP* genes from the “SFS” were designated *AsTCP001* to *AsTCP083*, while genes from the “Sang” and “OT3098v2” retained their respective genomic gene IDs.

### 2.3 Phylogenetic analysis of AsTCP gene family

DNAman was used to compare selected TCP amino acid sequences and construct a phylogenetic tree for oat TCPs, along with wheat, rice, and *A. thaliana* using MEGA11, with 1,000 bootstrap replicates. The phylogenetic tree was further refined using the Evolview online tool (https://www.evolgenius.info/evolview/#/treeview) to enhance visualization and clarity (He et al., 2016).

### 2.4 Analysis of the structure, physicochemical properties and promoter sequence of AsTCP gene

Conserved motifs in the oat *TCP* gene family were predicted using the MEME suite (https://meme-suite.org/meme/), with the number of predicted motifs set to 10 (Bailey et al., 2009). TCP protein structural domains were identified via the NCBI Conserved Domain Database (CDD) (https://www.ncbi.nlm.nih.gov/cdd/). Gene structure analysis was conducted using the online tool GSDS 2.0 (http://gsds.cbi.pku.edu.cn/). Visualization of motifs, domains, and gene structures was carried out using TBtools (v2.042) software (Chen et al., 2020). Subcellular localization was predicted using WoLF PSORT (https://wolfpsort.hgc.jp/) and the Molecular Bioinformatics Center (MBC) website (http://cello.life.nctu.edu.tw/) (Horton et al., 2007). Oat *TCP* family amino acid sequences were analyzed for molecular weight and isoelectric point (pI) using the Expasy website (https://web.expasy.org/compute_pi/) (Artimo et al., 2012). Cis-acting elements within 2 kb upstream of the start codon of oat *TCP* genes were analyzed using the PlantCARE online tool (https://bioinformatics.psb.ugent.be/webtools/plantcare/html/) (Lescot et al., 2002).

### 2.5 Chromosomal localization, covariance and interaction network analysis of AsTCP gene

Chromosomal localization of oat *TCP* genes was performed using TBtools software (Chen et al., 2020). Nonsynonymous (Ka) and synonymous (Ks) substitution rates were calculated for oat *TCP* genes using TBtools, and the Ka/Ks ratio was computed to assess evolutionary pressures influencing gene trends (>1 indicates positive selection, = 1 neutral selection, <1 purifying selection) (Chen et al., 2020). Duplication events of *AsTCP* genes were analyzed using the Multicollinearity Scanning Toolkit (MCScanX) with default parameters (Wang et al., 2012). The Dual Synteny Plotter tool within TBtools was employed to visualize synteny relationships of oat *TCP* genes with those from rice, maize, wheat, two-spike phragmites, and knapweed genomes (Chen et al., 2020). STRING (https://cn.string-db.org/) was employed to predict interacting proteins using Arabidopsis as the reference species. Additionally, psRNATarget (https://www.zhaolab.org/psRNATarget/analysis) was used to predict the miRNAs targeting AsTCP proteins (Dai et al., 2018). All results were visualized using Cytoscape 3.10.0 software.

### 2.6 Transcriptome data analysis

Transcriptome data related to silicon-mediated drought stress alleviation and salt stress in oats were sourced from the public NCBI (https://www.ncbi.nlm.nih.gov/sra/?term=Oat) (data number SRP237902, SRP093940) (Bray et al., 2016). FPKM values (log2 transformed) were used to analyze the expression of *TCP* family genes under the 2 treatments, and heatmaps were drawn using the Heatmap program of TBtools (Chen et al., 2020).

### 2.7 Real-time fluorescent quantitative PCR assay

Total RNA extraction from plants was performed using the Transzol Up Plus kit from Beijing All Style Gold. The first strand of cDNA was synthesized via reverse transcription, following the instructions provided with the PrimeScript RT kit from Takara. Quantitative PCR primers for *AsTCP021*, *AsTCP025*, *AsTCP033*, *AsTCP044*, *AsTCP053,* and *AsTCP058* were designed using Primer 5.0 software (Supplementary Table S1) and synthesized by Beijing Liuhe Huada Gene Science and Technology Co. Oat β-Actin was used as the internal reference gene (Zhang, 2023). The expression level of each *AsTCP* gene was quantified using the 2^-(ΔΔCt)^ method. The PCR reaction conditions were as follows: initial denaturation at 95°C for 5 min, followed by 40 cycles of denaturation at 95°C for 15 s, annealing at 58°C for 20 s, and extension at 72°C for 20 s.

## 3 Results

### 3.1 Identification of members of AsTCP gene family

Using the genomic data of oat cultivars “SFS”, “Sang”, and “OT3098v2”, we identified 83, 65, and 30 members of the *AsTCP* gene family, respectively (Supplementary Tables S2, S3). In “SFS” and “Sang”, the amino acid sequences of 34 AsTCPs showed 100% identity, and those of 13 AsTCPs had over 90% identity. In “SFS” and “OT3098v2”, the amino acid sequences of 16 AsTCPs exhibited 100% identity, and those of 5 AsTCPs had over 90% identity. In “Sang” and “OT3098v2”, the amino acid sequences of 16 AsTCPs presented 100% identity, and those of 6 AsTCPs had over 90% identity. The “OT3098v2” contained three unique *AsTCP* genes (*AVESA.00001b.r1.3Cg0000521.1*, *AVESA.00001b.r1.5Ag0002232.1*, *AVESA.00001b.r1.2Dg0002764.1*). The “Sang” had four unique *AsTCP* genes (*AVESA.00010b.r2.2DG0346510.1*, *AVESA.00010b.r2.2DG0402990.1*, *AVESA.00010b.r2.6DG1166000.1*, and *AVESA.00010b.r2.7AG1210110.1*). The “SFS” had 22 unique *AsTCP* genes (*AsTCP020* to *AsTCP022*, *AsTCP033*, *AsTCP035*, *AsTCP040*, *AsTCP045*, *AsTCP056*, *AsTCP064*, *AsTCP065*, *AsTCP068*, *AsTCP070*, *AsTCP072*, *AsTCP075* to *AsTCP083*). Analysis of the amino acid sequence lengths of *AsTCP* genes in the “SFS” showed substantial variation, ranging from 71 to 609 amino acids (aa). The shortest protein, *AsTCP081*, comprised 71 aa, whereas the longest protein, *AsTCP037*, comprised 609 aa. The molecular weights of the 83 *AsTCP* proteins varied from 7954.97 to 64420.89 Da, and their isoelectric points (pI) ranged from 4.3 to 10.78 (Supplementary Table S4). Subcellular localization analysis indicated that 72 (86.75%) of the *AsTCP* proteins were localized in the nucleus. The remaining proteins were distributed as follows: five in the extracellular region, two at the plasma membrane, two in the cytoplasm, one in the chloroplast, and one in the mitochondrion (Supplementary Table S4).

### 3.2 Phylogeny and classification of the oat AsTCP gene family

Phylogenetic analysis of TCP proteins from oat, *A. thaliana*, rice, and wheat revealed that the 83 AsTCP proteins encoded by the “SFS” could be classified into three subfamilies: class I PCF, class II CYC/TB1, and CIN. Specifically, 40 proteins were categorized under the PCF subfamily, 13 under CYC/TB1, and 30 under CIN (Supplementary Figure S1A). In the “Sang”, 37 AsTCP proteins were classified as PCF, 10 as CYC/TB1, and 18 as CIN (Supplementary Figure S1B). Similarly, the “OT3098v2” showed 16 proteins in the PCF subfamily, 3 in CYC/TB1, and 11 in CIN (Supplementary Figure S1C). In the evolutionary relationship of *TCP* genes among oat, wheat, rice and Arabidopsis, the *TCP* genes in oat have a closer phylogenetic relationship with the *TCP* genes in wheat. A total of 14 *AsTCP* genes were found in the hulled and naked oat varieties “SFS”, “Sang” and “OT3098v2”, and the amino acid sequences encoded by them showed complete consistency (Supplementary Tables S2, S3). Within the oat genome, there are also different evolutionary relationships among members of the *TCP* gene family. Most oat *TCP* genes, such as *AsTCP024*/*AsTCP023*/*AsTCP025*, which are homologous genes distributed in subgroups A/C/D, are the closest in evolution, and these three genes are all distributed in the three oat genomes. A small number of oat *TCP* genes, such as *AsTCP015*/*AsTCP017*/*AsTCP018* show a closer relationship to *AsTCP021*/*AsTCP020*/*AsTCP022*, *AsTCP001*/*AsTCP003*/*AsTCP002* with *AsTCP009*/*AsTCP008*/*AsTCP010*, *AsTCP043*/*AsTCP035* with *AsTCP049*/*AsTCP048*/*AsTCP050*, and *AsTCP013*/*AsTCP012* with *AsTCP014*/*AsTCP005*. These types of partially homologous gene families have similar evolutionary relationships (Supplementary Figure S1; Supplementary Table S2).

Analysis of amino acid sequences revealed that 65, 59, and 27 AsTCP proteins in the “SFS”, “Sang”, and “OT3098v2”, respectively, possessed a complete helix-loop-helix (bHLH) structure, indicating a high conservation of the TCP structural domain in oats. Notably, the basic region within the bHLH of CYC/TB1 and CIN subfamilies contained a bidirectional nuclear localization signal (NLS), crucial for protein translocation to the nucleus. In contrast, the PCF subfamily exhibited a partial NLS in its basic region (Supplementary Figure S2A). These sequence differences likely contribute to the observed conservation pattern within the PCF subfamily, and suggest functional divergence between the subfamilies. Furthermore, variations were observed in the basic region of the bHLH domain. The CYC/TB1 and CIN subfamilies have four additional amino acids compared to the PCF subfamily. Specific AsTCP proteins, such as AsTCP69 and AsTCP71 in both “SFS” and “Sang”, exhibited amino acid deletions in the basic region (Supplementary Figures S2A, B). Additionally, 16, 3, and 3 AsTCP proteins in “SFS”, “Sang”, and “OT3098v2”, respectively, showed deletions spanning the entire bHLH domain (Supplementary Figure S3).

### 3.3 AsTCP conserved motifs, structural domains and gene structure

**FIGURE 1:**
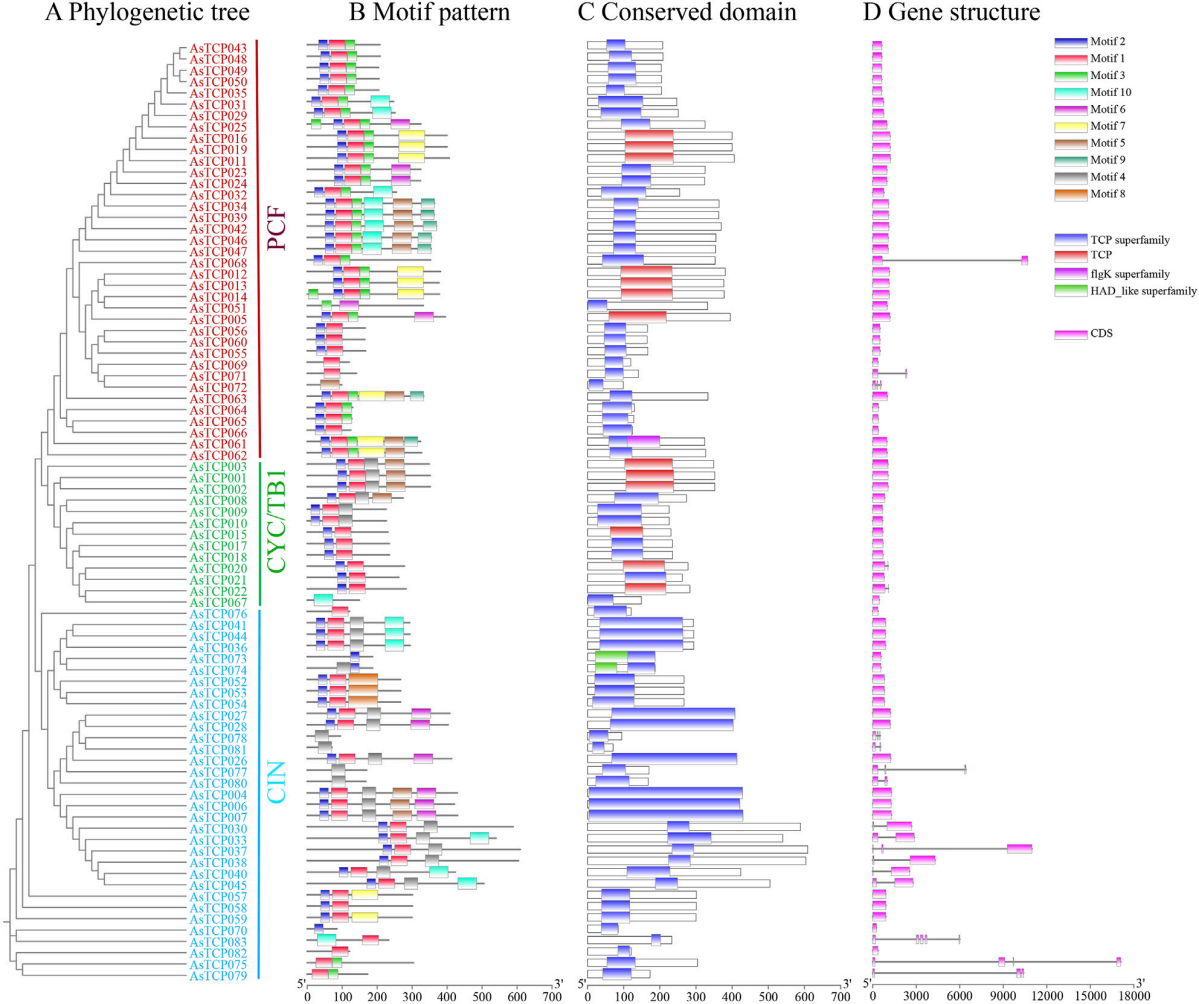
Phylogenetic analysis, motif patterns, conserved domains, and gene structure of TCP genes in the “SFS”. **(A)** Neighbor-Joining tree of oat TCP proteins; **(B)** Motif patterns, with motifs numbered 1-10 and represented by different colored boxes; **(C)** Conserved domains identified in the oat TCP proteins; **(D)** Gene structure representation, where CDS and introns are represented with pink boxes and black lines respectively.

The phylogenetic tree results show that the 83 AsTCP genes are divided into three subfamilies: PCF, CIN, and CYC/TB1 (Figure 1A). Conservative base sequencing analysis identified 10 motifs present in the 83 AsTCP proteins (Supplementary Table S5). Several *TCP* genes exhibited deletions within the bHLH structural domain: *AsTCP051*, *AsTCP067*, *AsTCP072*, *AsTCP077*, *AsTCP080*, *AsTCP078*, and *AsTCP081* lacked Motif 1 and Motif 2. While *AsTCP070*, *AsTCP073*, and *AsTCP074* were missing Motif 1 (Figure 1B). Similarly, the *AsTCP* gene of “Sang” (*AVESA.00010b.r2.5CG0872470.1*, *AVESA.00010b.r2.6CG1108510.1*) and the AsTCP gene of “OT3098v2” (*AVESA.001b.r1.2Dg0002764.1*, *AVESA.001b.r1.5Ag0002232.1*,*AVESA.00001b.r1.3Cg0000521.1*) is also in a similar situation (Supplementary Figures S4, S5). Genes within the same subfamily exhibited similar motif compositions, indicating conserved motif types and distributions among closely related genes. Motif 4 is specific to the Class II (CYC/TB1 and CIN subfamilies), Motif 8 is specific to the CIN subfamily, and Motif 9 is specific to the PCF subfamily. In addition to the TCP structural domain, *AsTCP061*, *AsTCP073*, and *AsTCP074* also contained two additional structural domains, flgK and HAD (Figure 1C). Gene structure analysis revealed that among the 18 *AsTCP* genes analyzed, intron numbers ranged from 1 to 4, with 78% of genes being intronless. Notably, the CIN subfamily exhibited the highest intron count among *AsTCP* genes (Figure 1D). Intronless *AsTCP* genes accounted for 65% and 40% of the total in the “Sang” and “OT3098v2”, respectively, significantly influencing the untranslated region (UTR) annotations of *AsTCP* genes (Supplementary Figures S4, S5). Additionally, differences in intron lengths within genes of the same subfamily contributed to significant variations in gene lengths.

### 3.4 Prediction of cis-acting elements in the promoter of AsTCP gene family

**FIGURE 2:**
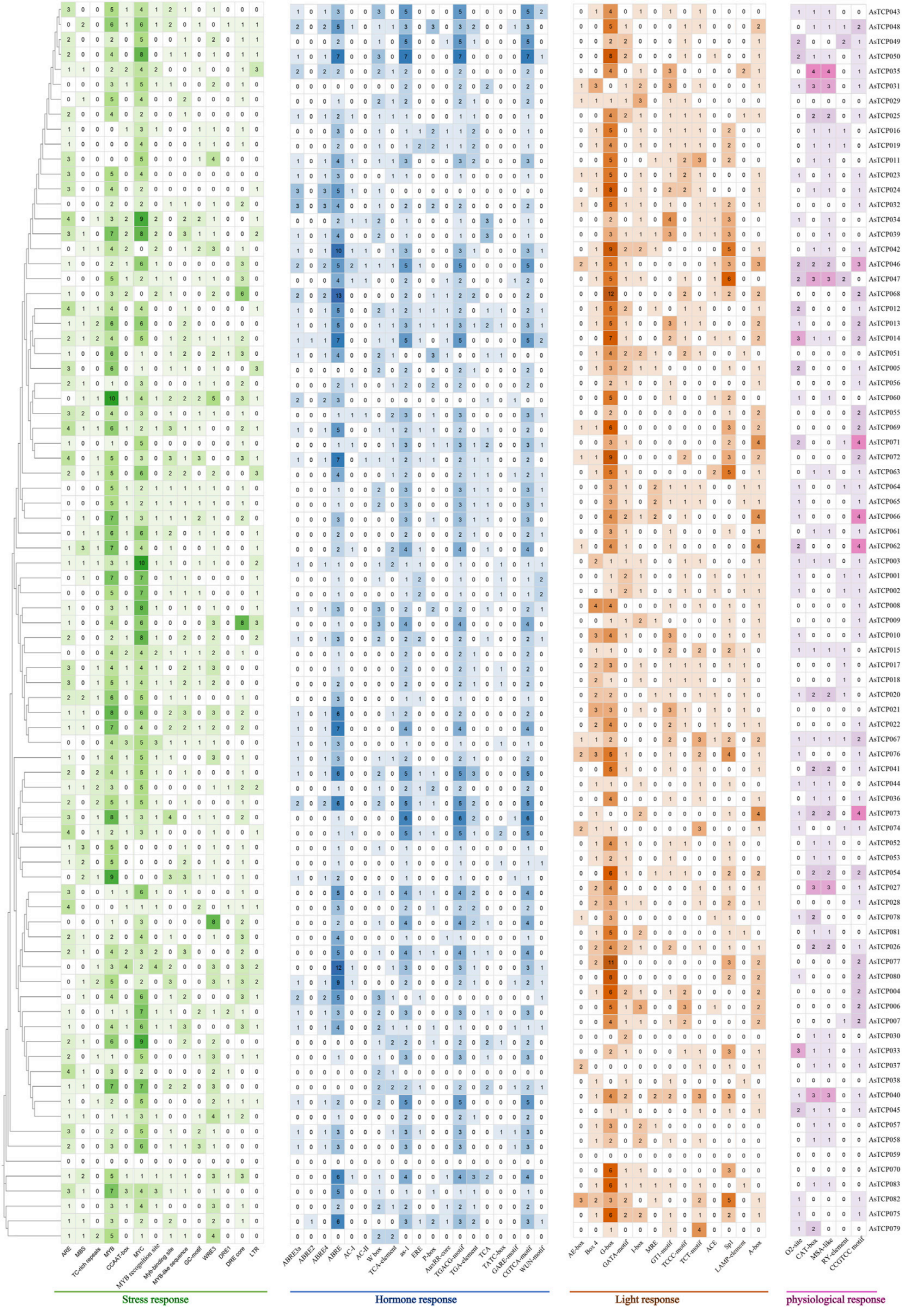
Cis-acting elements on promoters of oat *TCP* genes. ARE, cis-acting regulatory element essential for the anaerobic induction; MBS, MYB binding site involved in drought-inducibility; TC-rich repeats, cis-acting element involved in defense and stress responsiveness; CCAAT-box, MYBHv1 binding site; GC-motif, enhancer-like element involved in anoxic specific inducibility; LTR, cis-acting element involved in low-temperature responsiveness; ABRE, cis-acting element involved in the abscisic acid responsiveness; TCA-element, cis-acting element involved in salicylic acid responsiveness; P-box and GARE-motif, gibberellin-responsive element; AuxRR-core, cis-acting regulatory element involved in auxin responsiveness; TGACG-motif, cis-acting regulatory element involved in the MeJA-responsiveness; TGA-element, auxin-responsive element; TATC-box, cis-acting element involved in gibberellin-responsiveness; WUN-motif, wound-responsive element; AE-box, part of a module for light response; Box 4, part of a conserved DNA module involved in light responsiveness; G-box, cis-acting regulatory element involved in light responsiveness; GATA-motif, I-box, TCCC-motif, TCT-motif, and LAMP-element, part of a light responsive element; MRE, MYB binding site involved in light responsiveness; GT1-motif and Sp1, light responsive element; ACE, cis-acting element involved in light responsiveness; A-box, cis-acting regulatory element/sequence conserved in alpha-amylase promoters; O2-site, cis-acting regulatory element involved in zein metabolism regulation; CAT-box, cis-acting regulatory element related to meristem expression; MSA-like, cis-acting element involved in cell cycle regulation; RY-element, cis-acting regulatory element involved in seed-specific regulation.

A total of 4 types of elements, including 51 different cis-acting elements, were discovered in the promoter region of the *AsTCP* gene in “SFS” (Supplementary Table S6). 19 hormone response elements were widely distributed in the promoter regions of the *AsTCPs* (Figure 2), with the highest number of ABREs attributed to abscisic acid response elements, accounting for about 21% of the total number of hormone elements. In addition, the promoter regions harbored 14 types of stress-responsive elements, including those responsive to drought, endosperm-specific expression, low-temperature, cell cycle regulation, meristem expression, maize protein metabolism regulation, and defense stress responses (Figure 2). MYC and MYB elements, crucial for environmental adaptation, were particularly abundant, accounting for approximately 23.7% and 23.2% of total stress-responsive elements, respectively. 13 types of light-responsive elements were also identified, with G-box elements (Figure 2), which can bind MYC proteins, being the most prevalent at about 34.2% of all light-responsive elements. Furthermore, 5 types of physiological response elements were found, widely distributed across *AsTCP* gene promoters (Figure 2). The CCGTCC motif was the most abundant, comprising approximately 30.6% of all physiological response elements. The types of elements in the promoter regions of the AsTCP gene in “SFS”, “Sang” and “OT3098v2” are similar (Supplementary Figures S6– S8).

### 3.5 Comparative analysis of chromosome localization and covariance

**FIGURE 3:**
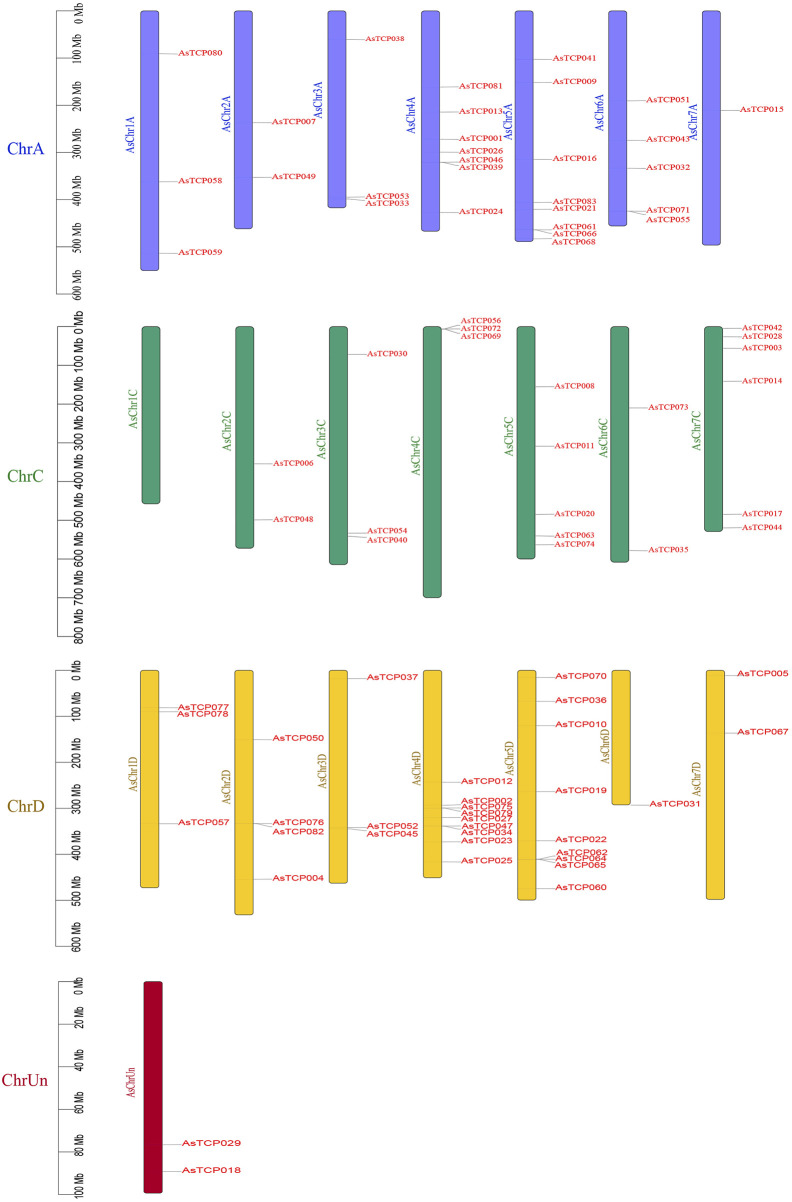
Chromosomal location of the *AsTCP* genes in the “SFS”.

In the “SFS”, 21 *AsTCP* genes were localized on chromosome A, 29 on chromosome C, 31 on chromosome D, and 2 on Un (Figure 3). In the “Sang”, 22 *AsTCP* genes were localized on chromosome A, 16 on chromosome C, 23 on chromosome D, and 4 on Un (Supplementary Figure S9A). For the “OT3098v2”, 12 *AsTCP* genes were localized on chromosome A, 5 on chromosome C, 11 on chromosome D, and 2 on Un (Supplementary Figure S9B). Generally, the distribution of *AsTCP* genes across the A, C, and D subgenomes was relatively even, with the highest number of *AsTCP* genes found on subgenomes 4D and 5D, each containing 9 genes. Notably, only one gene was found on subgenome 6D, and none were present on 1C.

**FIGURE 4:**
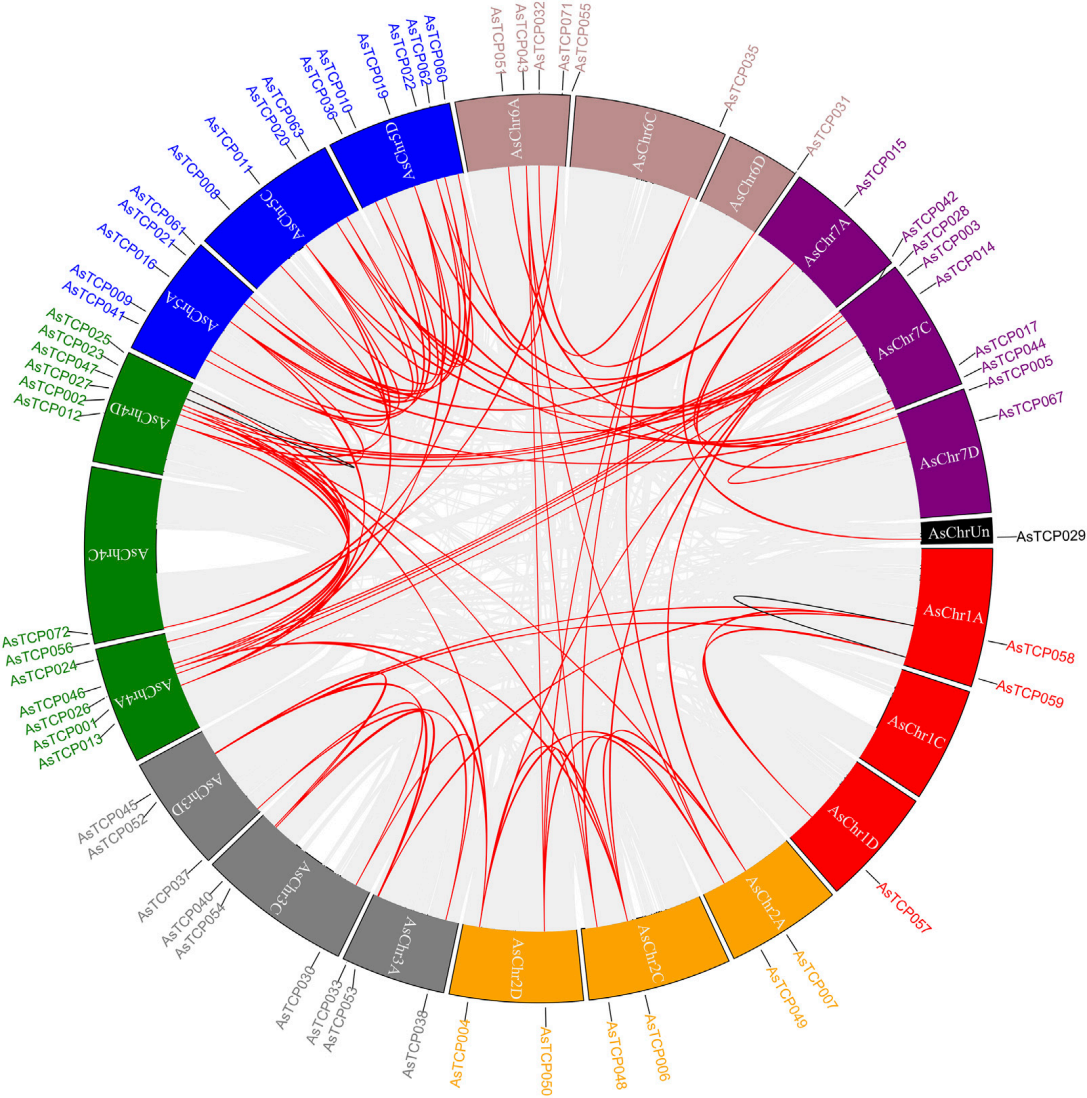
Synteny analysis of *AsTCP* genes in oat. Red colored lines indicate segmental duplication gene pairs, black lines indicate tandem duplication pairs. Chromosomes 1 red, 2 orange, 3 gray, 4 green, 5 blue, 6 maroon, 7 purple, Un black.

Gene duplication event analysis revealed 89, 73, and 20 homologous pairs of *AsTCP* gene family members in the “SFS”, “Sang”, and “OT3098v2”, respectively. Among these, two pairs (*AsTCP058*/*AsTCP059* and *AsTCP023*/*AsTCP025*) were identified as tandem duplications across all three genomes, while the remaining 87, 71, and 18 pairs were segmental duplications (Figure 4; Supplementary Figure S10). Notably, 89 homologous gene pairs in the “SFS” exhibited Ka/Ks < 1, indicating that these genes were primarily subject to purifying selection, with no pairs evolving under strong positive selection (Ka/Ks > 1) post-duplication (Supplementary Table S7). These findings suggest that oat *TCP* genes underwent both segmental and tandem duplications, with segmental duplication being the predominant mode, and two pairs of tandem duplications remaining stable across the three oat genomes.

**FIGURE 5:**
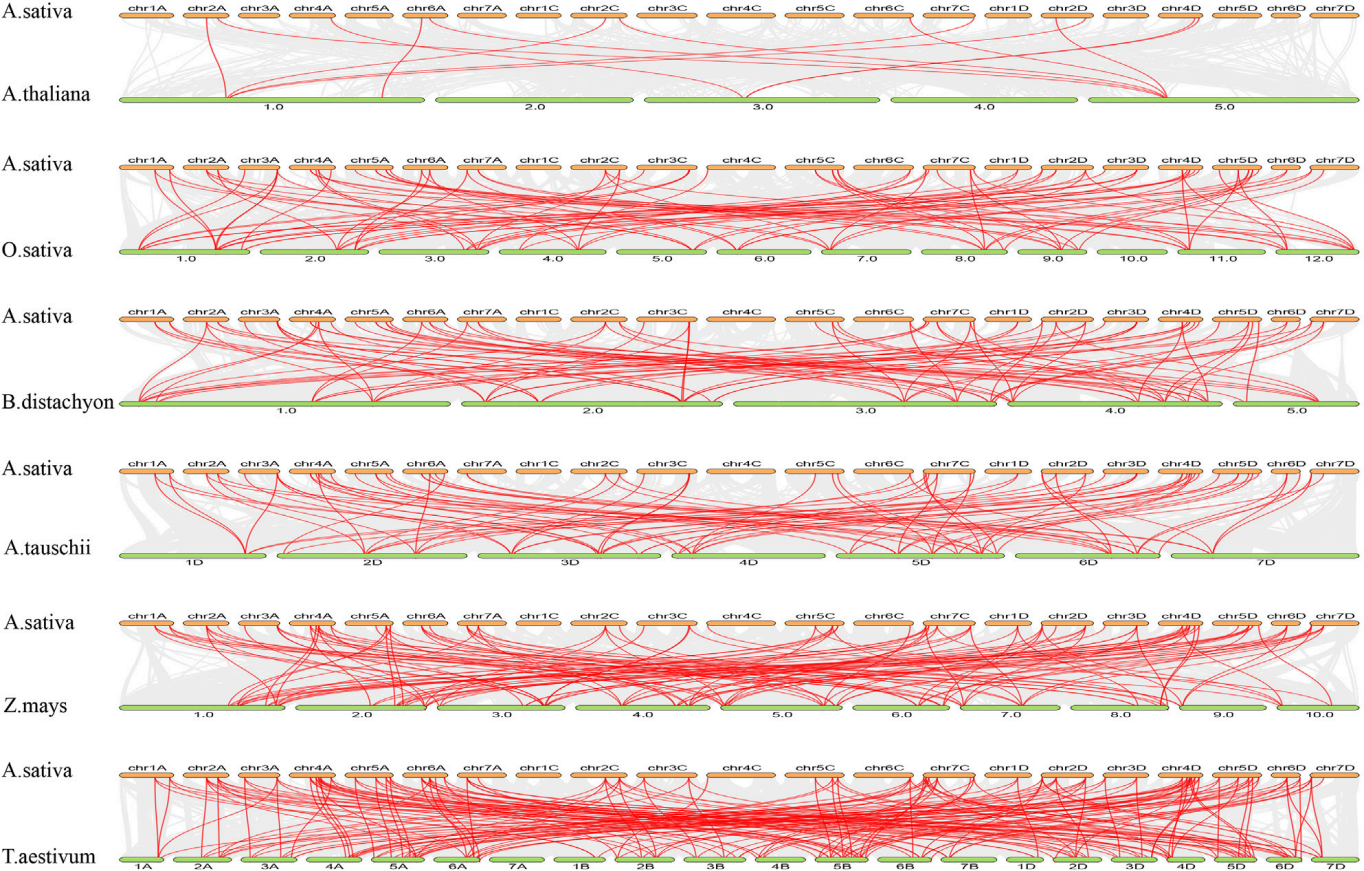
Synteny analysis of *AsTCP* genes between oat and six representative plant species. The gray lines show colinear blocks in the genomes of oat with other plants, while the red line highlights the colinear *TCP* pairs. The species name with the prefixes “*A.sativa”*, “*A.thaliana”*“*O. sativa”* ‘*B.distachyon”*, “*A*.*tauschii”*, “*Z.mays”* and “*T. aestivum”* indicate *Avena sativa*, *Arabidopsis thaliana*, *Oryza sativa*, *Brachypodium distachyon*, *Aegilops tauschii*, *Zeamays*, and *Triticum aestivum*, respectively.

Comparative collinearity analysis between oat (“SFS”) and other species, including *A. thaliana*, rice, *Brachypodium*, *Setaria*, maize, and wheat, revealed the highest *TCP* gene homology with wheat, comprising 175 collinear pairs between 54 *AsTCPs* and *54 TaTCPs*. Conversely, the lowest homology was observed with *A. thaliana*, where 14 *AsTCPs* exhibited collinearity with 4 *AtTCPs*, resulting in 15 collinear pairs (Figure 5; Supplementary Table S8). 42 *AsTCP* genes in the oat genome showed collinearity with *TCP* genes in all other species, excluding Arabidopsis. 9 out of the 12 *AsTCP* genes collinear with Arabidopsis were also part of these 42 genes (Supplementary Table S8). These duplicated *AsTCP* genes, exhibiting collinearity with other species, likely participated more frequently in gene duplication events, playing significant roles in the evolutionary trajectory of oats.

### 3.6 Protein interaction network and miRNA prediction of AsTCP gene

**FIGURE 6:**
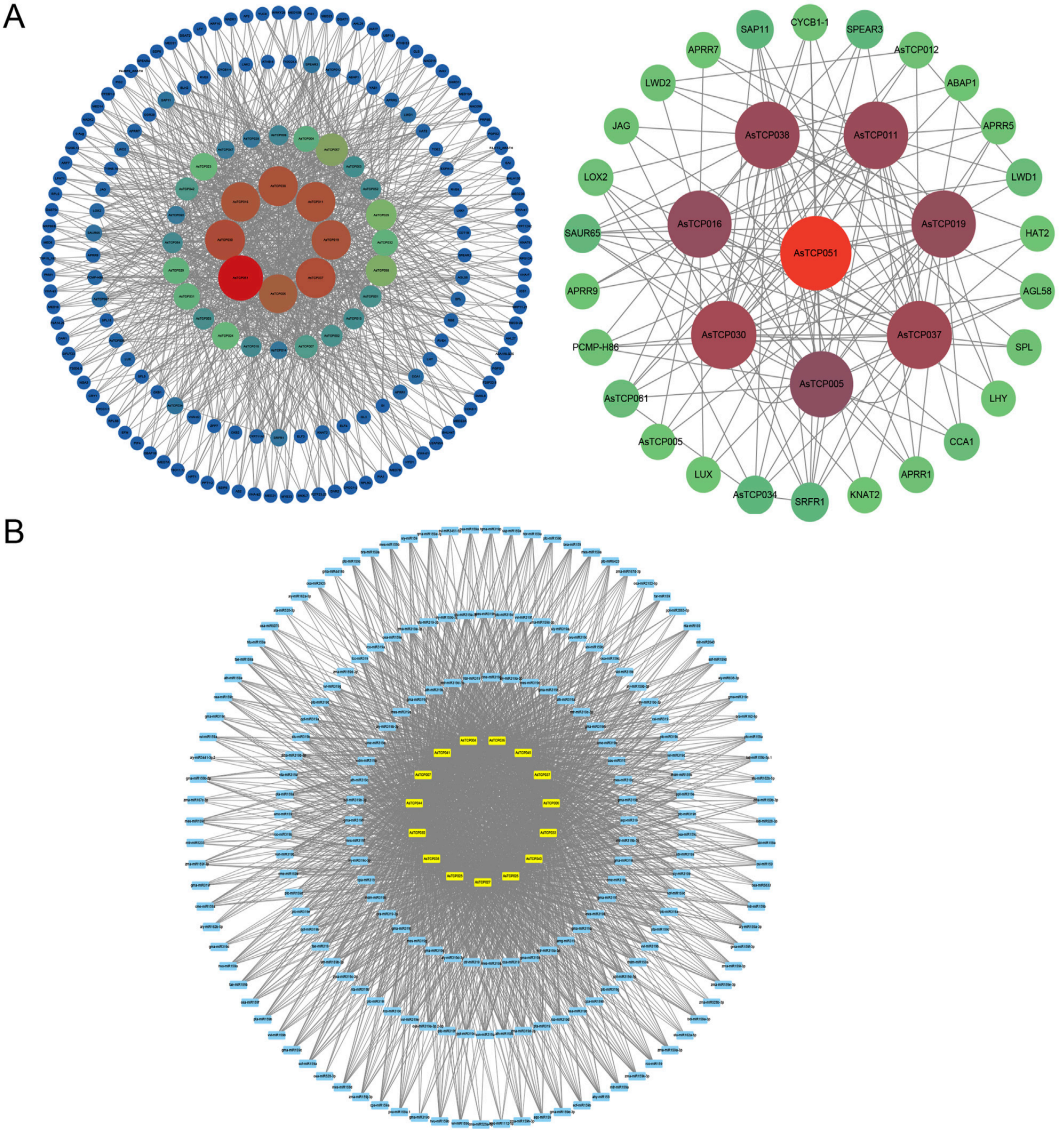
Protein-Protein Interaction Network and *miRNA* Prediction for *AsTCP* Genes. **(A)** The protein interaction network based on the Arabidopsis homologue AsTCP protein; **(B)** The relationship between members of the *AsTCP* gene family and *miRNAs*.

The predicted protein interaction relationships of *AsTCP* genes as well as miRNA realisation results were visualised by Cytoscape software. The results showed that 35 AsTCP proteins were predicted to have interaction relationships with other proteins, among which, 8 proteins, AsTCP005, AsTCP016, AsTCP019, AsTCP030, AsTCP037, AsTCP038, and AsTCP051, had a higher number of interacting proteins. Among those interacting proteins, SAUR65, SRFR1, APRR1, SAP11, and CYCB1-1 have higher chance to interact with these 8 AsTCP proteins as mentioned above (Figure 6A). When predicting the upstream regulator *miRNAs* of *AsTCP* genes, 197 upstream regulator *miRNAs* were found for 15 *AsTCP* genes, including *AsTCP004*, *AsTCP006*, *AsTCP007*, *AsTCP026*, and *AsTCP027*. When the expectation value was less than 2.5, all the upstream regulatory factors were *miR319* (Figure 6B; Supplementary Table S9). Therefore, *miR319* may play an important regulatory role in the *AsTCP* gene family.

### 3.7 Expression profiles of AsTCP genes under abiotic stresses

**FIGURE 7:**
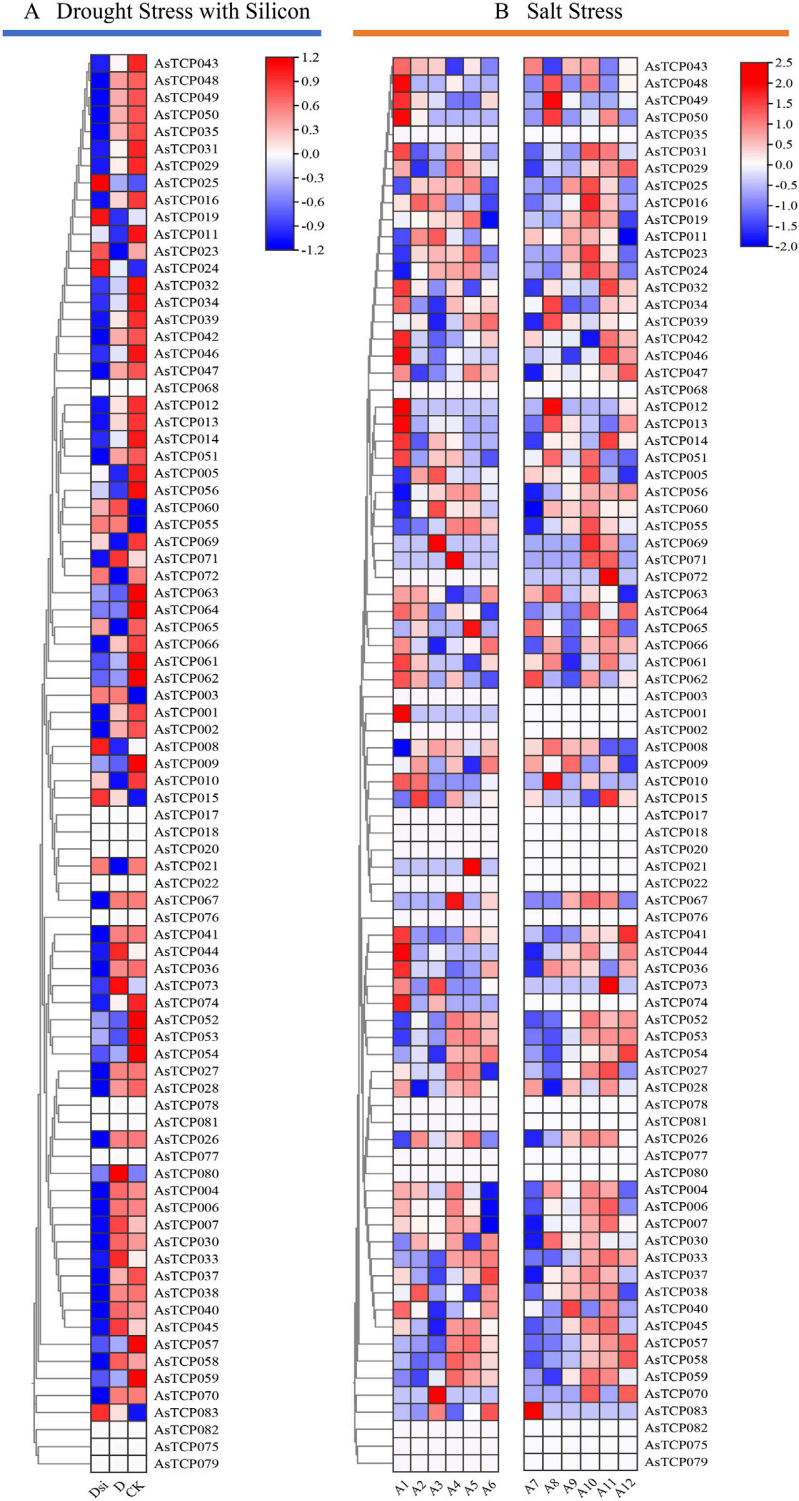
The expression patterns of 83 *AsTCP* genes stress levels are based on RNA-seq data.**(A)** Analysis of *TCP* gene expression induced by exogenous silicon addition Under drought conditions,D (Drought stress),Dsi (Adding exogenous silicon under drought stress); **(B)** Expression analysis of *TCP* genes was conducted on Huazao-2 under salt stress at 0 h (A1), 2 h (A2), 4 h (A3), 8 h (A4), 12 h (A5), and 24 h (A6), and on Hanyou-5 at 0 h (A7), 2 h (A8), 4 h (A9), 8 h (A10), 12 h (A11), and 24 h (A12) under salt stress.

Given the critical role of the *TCP* gene family in regulating plant stress resistance, the expression patterns of 83 *AsTCP* genes under silicon-mediated drought stress alleviation and salt stress treatments were analyzed (Supplementary Tables S10, S11). 83 *AsTCP* genes (excluding *AsTCP068*, *AsTCP020*, *AsTCP076*, *AsTCP078*, *AsTCP077*, *AsTCP082*, *AsTCP075*, *AsTCP079*) had significant responses to drought and salt stresses (Figure 7). Under drought treatment, *AsTCP005*, *AsTCP010*, *AsTCP011*, *AsTCP021*, *AsTCP053*, *AsTCP056*, *AsTCP065*, *AsTCP069*, and *AsTCP072* were significantly downregulated, whereas in the control group, these genes were generally significantly upregulated. On the other hand, *AsTCP007*, *AsTCP033*, *AsTCP044*, *AsTCP045*, *AsTCP071*, *AsTCP073*, and *AsTCP080* were significantly upregulated under drought treatment but significantly downregulated silicon-mediated drought stress alleviation. These genes generally exhibited low expression levels in the control group (Figure 7A). These results suggest that the aforementioned genes may play key roles in responding to drought stress. Under short-term salt stress, different oat varieties exhibited varying sensitivities to salt stress. There were significant differences in the timing and expression patterns of some *AsTCP* genes between the two cultivars, Huazao-2 and Hanyou-5. In addition, the majority of *AsTCPs* were significantly upregulated at 8 h and 12 h of salt stress (Figure 7B).

### 3.8 Expression patterns of AsTCP genes in abiotic stresses

**FIGURE 8:**
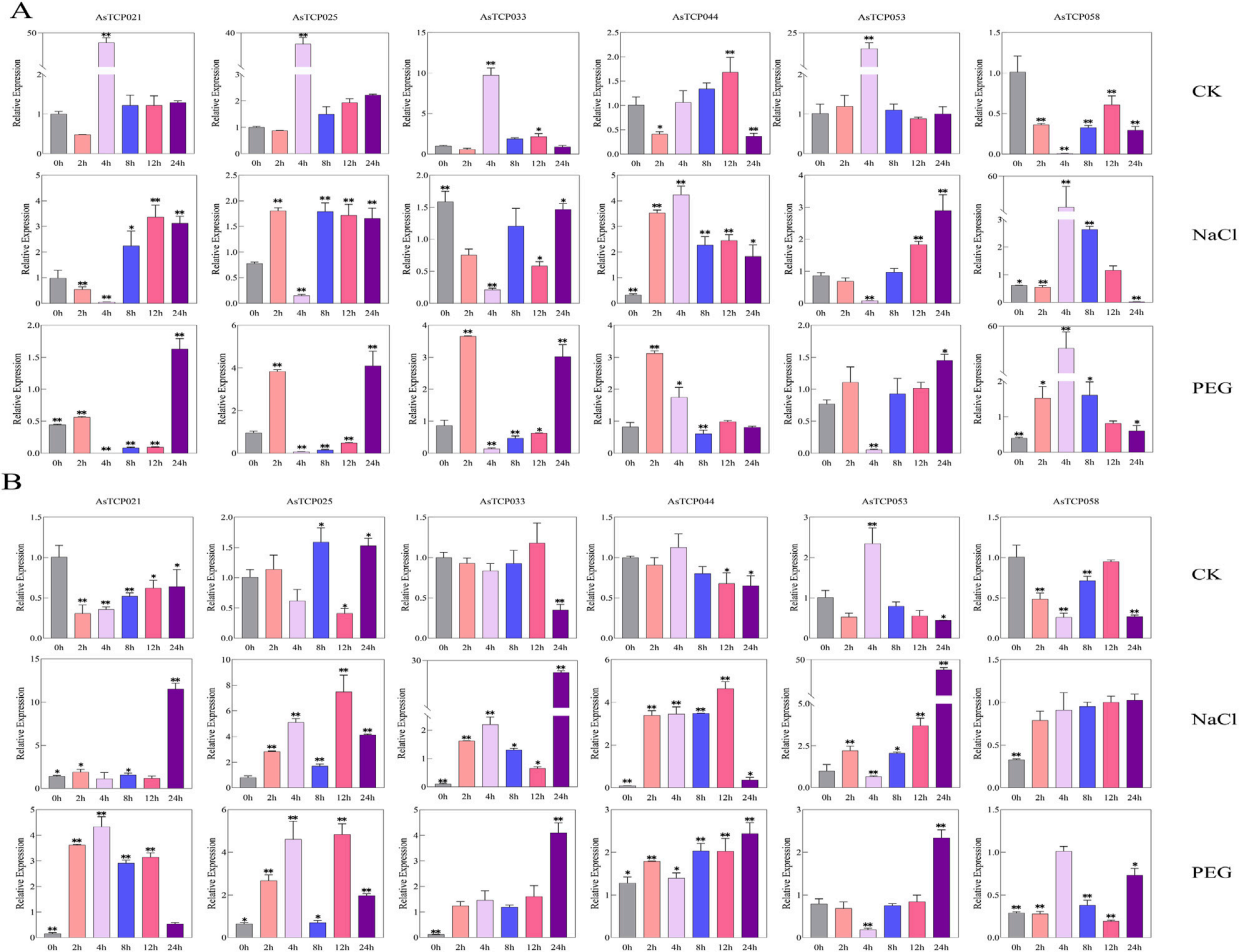
Expression of 6 *AsTCP* Genes Under Untreated (CK), Drought (PEG6000), and Salt (100 mM NaCl) Conditions. **(A)** Expression of six *AsTCP* genes in NY6; **(B)** Expression of six *AsTCP* genes in QY1. Explanations: CK Group: *AsTCP* genes expression with 0 h as the control, NaCl and PEG Groups. *AsTCP* genes expression with the corresponding CK time point as the control. Significant differences between treatment and control groups are indicated by * (LSD test, p < 0.05) and ** (p < 0.01).

To investigate the expression levels of *AsTCP* genes in different oat varieties under drought stress (20% PEG6000) and salt stress (100 mM NaCl), six stress-responsive genes were selected from the oat *TCP* expression profile for qRT-PCR analysis. Among them, *AsTCP021* and *AsTCP033* were identified in the “SFS”, *AsTCP044* and *AsTCP053* were identified in both the “SFS” and “Sang”, while *AsTCP025* and *AsTCP058* were identified in the “SFS”, “Sang” and “OT3098v2”. In NY6, *AsTCP044* and *AsTCP058* were significantly upregulated at 4 h under NaCl treatment, whereas *AsTCP021*, *AsTCP033*, and *AsTCP053* were significantly downregulated at 4 h (Figure 8A). In QY1, *AsTCP021*, *AsTCP033*, and *AsTCP053* were significantly upregulated at 24 h under NaCl treatment, while *AsTCP033*, *AsTCP044*, and *AsTCP058* were significantly downregulated at 0 h (Figure 8B). Comparison with transcriptome results from Huazao-2 and Hanyou-5 revealed that *AsTCP025*, *AsTCP033*, *AsTCP044*, and *AsTCP058* displayed similar expression trends at 0 h and 2 h across the four varieties. Furthermore, *AsTCP053* showed consistent expression trends in the four varieties, being downregulated at 0 h, 2 h, and 4 h, and upregulated at 8 h, 12 h, and 24 h, except for QY1, where it was upregulated at 2 h. In NY6, *AsTCP021*, *AsTCP025*, and *AsTCP053* were significantly upregulated at 24 h under PEG treatment, while *AsTCP021*, *AsTCP025*, *AsTCP033*, and *AsTCP053* were significantly downregulated at 4 h (Figure 8A). In QY1, *AsTCP033*, *AsTCP044*, and *AsTCP053* were significantly upregulated at 24 h under PEG treatment, while *AsTCP021* and *AsTCP033* were significantly downregulated at 0 h (Figure 8B). The qRT-PCR results for *AsTCP021* and *AsTCP053* in NY6 under drought stress from 0 h to 12 h were largely consistent with transcriptome results, both showing a trend of downregulation.

In both NY6 and QY1, the six *AsTCP* genes exhibited significant responses to both salt and drought stress, with the most notable changes observed at 0 h, 4 h, and 24 h. At 0 h under drought and salt stress, *AsTCP058* was significantly downregulated in both varieties. At 4 h, *AsTCP044* was significantly upregulated in both varieties, while *AsTCP053* was significantly downregulated. At 24 h, *AsTCP025*, *AsTCP033*, and *AsTCP053* were significantly upregulated in both varieties. Additionally, in the QY1 variety, *AsTCP033* and *AsTCP053* showed the most significant upregulation at 24 h under both drought and salt stress. The expression patterns of different genes at different time points varied significantly between the two varieties, particularly at 24 h, where the notable upregulation of some genes in QY1 reflected a stronger response to stress. These findings indicate that varietal differences in stress sensitivity significantly influence the expression patterns of these genes.

## 4 Discussion

*TCP* gene family members have been identified in various grass species, including 66 in wheat (Zhao et al., 2018), 46 in maize (Ding et al., 2019), 24 in Arabidopsis, and 28 in rice (Yao et al., 2007), as well as 20 in sorghum (Lei et al., 2021), 42 in willowherb (Huo et al., 2019), and 22 in orchardgrass (Wang C. et al., 2023). In this study, we identified 83, 65, and 30 *TCP* genes in the three oat genomes, respectively. Each of these *AsTCP* genes was specific to their corresponding oat genomes (Supplementary Table S2). As oats are heterozygous hexaploids, the presence of multiple gene copies within their genomes can be attributed to extensive replication and diversification during evolution (Song et al., 2015). The differences in the number of *TCP* genes among the three oat genomes may be attributed to several factors. Firstly, with the advancement of sequencing technologies and the availability of reference oat genomes, the genome assembly techniques and methods used in later sequencing projects have become more refined, allowing for greater sequencing depth. Additionally, different gene annotation methods and parameters can lead to variations in the number of identified genes (Tortuero et al., 2021). Secondly, transcription factors exist in various subtypes or alternative splicing forms, which may be identified as distinct genes in different genomes. Moreover, transcription factor families evolve rapidly, and events such as gene duplication, loss, or variation in different genomes can result in significant differences in gene copy numbers within these families (Adams and Wendel, 2005; Wang et al., 2018). Despite these differences, the *AsTCP* genes were classified into three subclasses: Class I (PCF), Class II (CIN), and (CYC/TB1) (Supplementary Figure S1). The high similarity between oat *TCP* genes and those in Arabidopsis, rice, and wheat suggests that *TCP* genes are highly conserved in plants (Martín-Trillo and Cubas, 2010). This further indicates that the *AsTCP* genes in oats evolved from a common ancestor within the Gramineae family, undergoing different modes of divergence in different lineages. In our study, we found that most of the *TCP* genes with amino acid deletions within the bHLH structure lacked Motif 1 and Motif 2, which are presumed to be key components of the TCP structural domain. Additionally, we observed that most oat *TCP* genes lacked intronic structures (Figure 1D and Supplementary Figures S4, S5). This may be related to the fact that intronless genes are often derived from horizontal gene transfer of intronless ancient prokaryotes, replication of existing intronless genes, or retrotranscription of intron-containing genes (Zou et al., 2011). Moreover, differences in conserved motifs and gene structures among subfamilies within the oat *AsTCP* gene family likely contribute to its functional diversity.

Members of the oat *TCP* family exhibit extensive variation in amino acid sequences, isoelectric points, relative molecular masses, and exon numbers, indicating their structural complexity and functional diversity (Xiao et al., 2018). Subcellular localization analysis revealed that most oat TCP proteins are localized in the nucleus and are also present in the extracellular, plasma membrane, cytoplasm, chloroplasts, and mitochondria (Supplementary Table S4). This distribution pattern may be associated with the broad range of roles that *TCP* genes play in plant growth and development. Transcription factors (TFs) specifically bind to cis-acting elements to regulate the expression of target genes (Zhao et al., 2021), and the diversity of these cis-acting sites determines the regulatory functions of TFs(Lei et al., 2021). Our analysis of promoter cis-acting elements found a variety of elements involved in hormone response, light response, and stress response were widely distributed in the promoters of the 83 *AsTCP* genes (Figure 2). This further highlights the involvement of *TCP* family genes in various biological processes, including photosynthesis, hormone regulation, growth and development, and stress response in plants (Ling et al., 2020; Ren et al., 2021; Xiong et al., 2022). In the oat genome, *TCP* genes exhibit a widespread phenomenon of multiple copies, such as *AsTCP024*/*AsTCP023*/*AsTCP025*, which are distributed in the A/C/D subgenomes. They demonstrate the closest evolutionary relationship among the three oat subgenomes. Additionally, *AsTCP001*/*AsTCP002* show a more similar evolutionary relationship to *AsTCP009*/*AsTCP008*/*AsTCP010*, as well as *AsTCP013*/*AsTCP012* to *AsTCP014*/*AsTCP015*. These genes are distributed in both the “SFS” and “Sang” (Supplementary Figure S1; Supplementary Table S2). These closely related genes in evolutionary terms likely share more similar sequences and structures, potentially indicating higher functional similarity (Peterson et al., 2010). Plants have undergone large-scale chromosome doubling events during evolution (Flagel and Wendel, 2009), and each replication or doubling of the genome leaves traces of loss, transfer, and recombination on the chromosomes (Peng et al., 2022). Our study revealed a direct correlation between the distribution of *TCP* genes in the three oat genomes and the lengths of the chromosomes, with none of the being located on chromosome 1C. Gene duplication analysis indicated that segmental duplications predominated in the oat *TCP* gene family, with two pairs of tandem duplication events consistently present in all three genomes (Figure 4 and Supplementary Figure S10). These findings suggest that segmental duplications contribute to the amplification and evolution of the oat *TCP* gene family. Interestingly, segmental duplications are also prevalent in Arabidopsis and rice, suggesting a common mechanism for *TCP* gene duplication in plant genomes (Liu et al., 2022). Genome-wide covariance analysis demonstrated that oat*s* had the highest number of covariant pairs with wheat and the fewest with Arabidopsis (Figure 5). This difference may be attributed to the genomic characteristics and evolution of oats, being a homozygous hexaploid plant that shares a closer evolutionary origin with wheat. *Arabidopsis thaliana*, a diploid dicotyledonous plant, evolved from a common ancestor shared with gramineous plants. However, the evolution of monocots predates that of dicots, and *TCP* genes may have undergone different modes of divergence between these two plant groups.

Oats are known for their high adaptability to harsh environments, and a gene’s function can often be inferred from its expression profile (Jewiss, 1972). Our analysis revealed that more than half of the *AsTCP* genes are involved in the response of oats to drought and salt stress (Figure 7). Similar results have been reported in canola (Brassica napus) (Wen et al., 2021), cotton (Yin et al., 2018), and orchardgrass (Huo et al., 2019). Studies have shown that *TCP* genes are involved in plant defense against abiotic stresses by regulating the expression of downstream genes. For example, rice *OsPCF2* regulates the downstream *OsNHX1* genes to enhance salt and drought tolerance (Almeida et al., 2017), whereas bamboo *PeTCP10* binds to the downstream *BT* gene to regulate drought tolerance (Liu et al., 2020). In our study, we observed temporal differences in the response of *TCP* genes to salt and drought stress in two oat varieties, with variations in expression patterns and degrees of response (Figure 7B). The same conclusions were drawn from the expression profiles of Huazao-2 and Hanyou-5 under salt stress. Specifically, in QY1, the expression of *AsTCP044*, *AsTCP058*, and *AsTCP033* was significantly downregulated at 0 h and 4 h after NaCl treatment (Figure 8B). In NY6, their expression was significantly downregulated at 0 h and 4 h but upregulated at 0 h after NaCl treatment (Figure 8A). Similarly, after PEG treatment, *AsTCP021* and *AsTCP053* were significantly downregulated and upregulated at 4 h and 24 h in NY6(Figure 8A), but downregulated at 0 h in QY1 (Figure 8B). These differences in expression may be attributed to the varying sensitivities of different oat varieties to the stress environment. Furthermore, at 0 h, 4 h, and 24 h of both stress treatments, the expression pattern of the *AsTCP053* gene was consistent in the two varieties, but there were differences in the degree of response to stress between them at 4 h and 24 h. Notably, at 24 h of drought and salt stress, the upregulation of the *AsTCP053* gene was more prominent in QY1 compared to NY6 Particularly under salt stress at 24 h, the upregulation of *AsTCP053* in QY1 was remarkably significant. Our study revealed that the expression of the *AsTCP053* gene exhibited an upregulation trend at 8 h, 12 h, and 24 h under salt stress treatment across four varieties, with expression levels gradually increasing over time. Prediction analysis indicated that the promoter region of *AsTCP053* is enriched with various cis-acting elements, including MYB and ARE. Under salt stress conditions, ARE element may interact with Nrf2-like transcription factors to activate genes related to antioxidant synthesis, thereby scavenging ROS accumulation (Zgorzynska et al., 2021). Meanwhile, the MYB element may function through the ABA signaling pathway by cooperating with MYB transcription factors to further activate downstream stress-responsive genes (Bo et al., 2015). The coordinated regulation of antioxidant and stress-responsive mechanisms mediated by ARE and MYB elements likely constitutes the key molecular basis for the dynamic regulation of *AsTCP053* under salt stress conditions. We hypothesize that the *AsTCP053* gene is a key regulator in oat response to salt stress, but currently, there is a lack of reports on its downstream genes in the salt stress pathway. We will focus on the exploration of the downstream gene functions of *AsTCP053* in our future research.

## 5 Conclusion

We analyzed the genome data of oats “SFS”, “Sang” and “OT3098v2” to identify *TCP* genes and perform bioinformatics analysis on them. Additionally, we selected six representative *AsTCP* genes to examine their expression patterns in hulled and hulless oats under drought and salt stress treatments.

1. In the genomes of the three oat varieties “SFS”, “Sang” and “OT3098v2”, we identified 83, 65, and 30 *TCP* genes, respectively, with some genes being unique to specific genomes.
2. The bHLH domain of some *AsTCPs* showed varying degrees of deletion, with Motif 1 and Motif 2 being key components of the bHLH domain. Only a small proportion of *AsTCP* genes in oats contain introns.
3. Segmental duplication is a common mechanism driving the expansion of the *AsTCP* gene family, with two pairs of tandemly duplicated *TCP* genes consistently present in the three oat genomes.
4. The genes *AsTCP021*, *AsTCP025*, *AsTCP033*, *AsTCP044*, *AsTCP053*, and *AsTCP058* are involved in the oat response to drought and salt stress, with significant gene expression observed at 0 h, 4 h, and 24 h. Due to differences in variety sensitivity to stress, the response time and gene expression patterns varied between the two oat varieties under the same stress conditions

## Data Availability

The original contributions presented in the study are included in the article/Supplementary Material, further inquiries can be directed to the corresponding author.
