# Supplementary material for: Genome-wide identification of oat TCP gene family and expression patterns under abiotic stress
Source: Front Genet. 2025 Feb 4;16:1533562. doi: 10.3389/fgene.2025.1533562 (PMC11832536; doi:10.3389/fgene.2025.1533562)
Supplement: Supplementary file 5 [file DataSheet1.pdf]

Supplementary Material

1 Supplementary Figures and Tables

1.1 Supplementary Figures

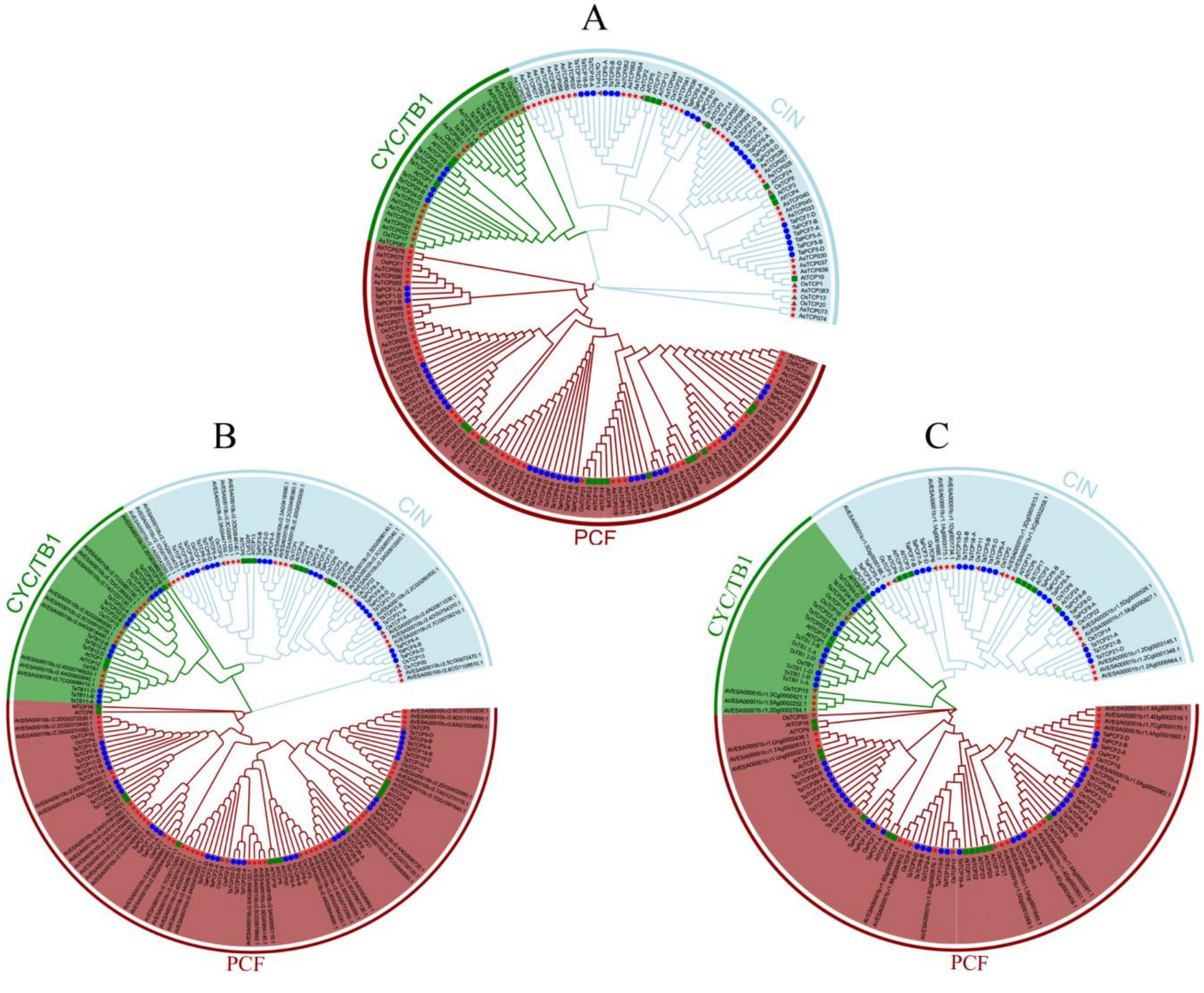

Supplementary Figure 1. Phylogenetic tree of TCP proteins in *Arabidopsis thaliana*, *Oryza sativa* (rice), *Triticum aestivum* (wheat), and *Avena sativa* (oat). Different colored arcs represent distinct groups or subgroups. Rectangles, triangles, circles, and stars denote *A. thaliana*, *O. sativa*, *T. aestivum*, and *A. sativa*, respectively. (A) The TCP proteins of oat are derived from the oat variety 'SFS'; (B) The TCP proteins of oat are derived from the oat variety 'Sang'; (C) The TCP proteins of oat are derived from the oat variety 'OT3098' .

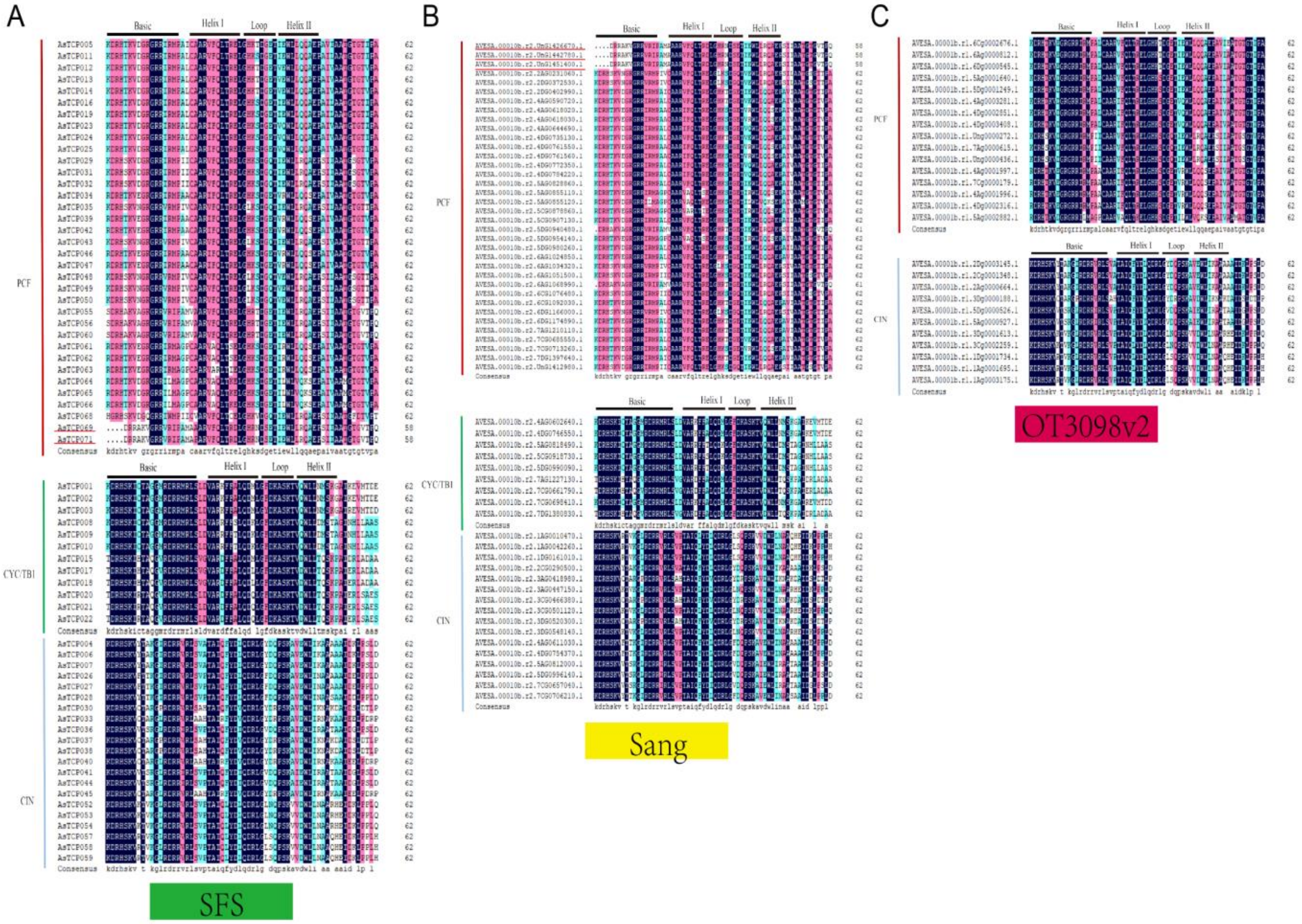

Supplementary Figure 2. Multiple sequence comparison of oat TCP proteins. Three colour box lines highlight residues conserved in all three TCP classes; red, residues conserved in the PCF class; green, residues conserved in the CIN class; and blue, residues conserved in the CYC/TB1 class. The basic, helix I, loop, and helix II regions are underlined in black at the top of the comparison. (A) The TCP proteins of oat are derived from the oat variety 'SFS'; (B) The TCP proteins of oat are derived from the oat variety 'Sang'; (C) The TCP proteins of oat are derived from the oat variety 'OT3098'.

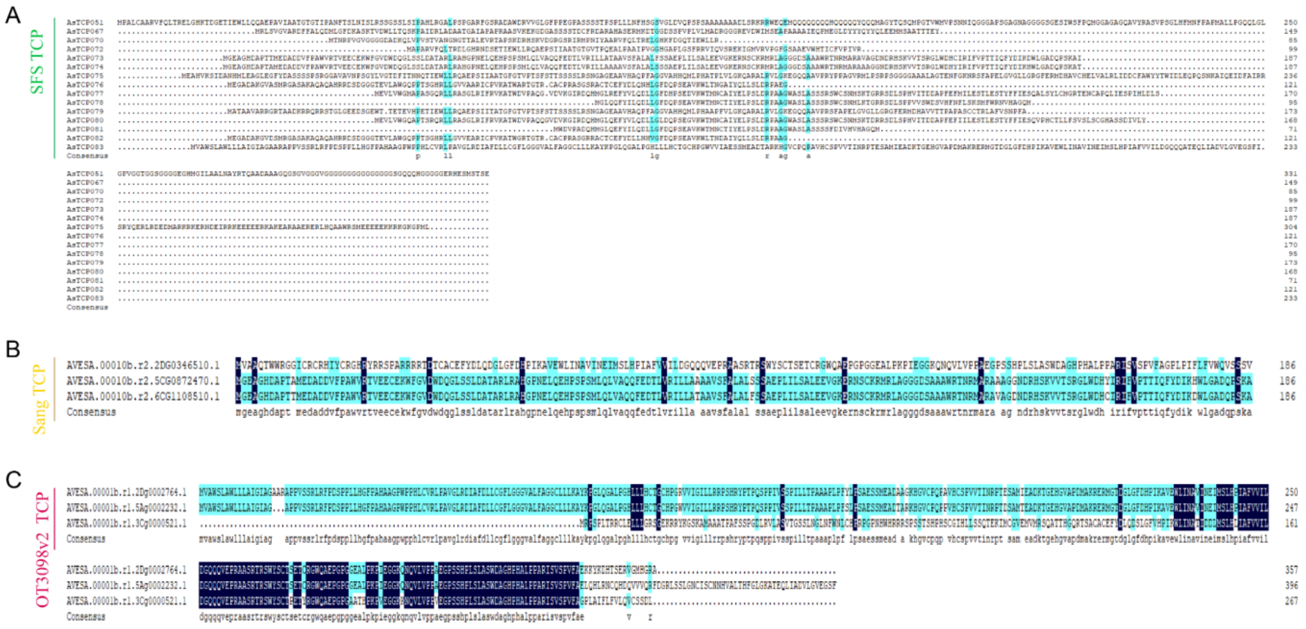

Supplementary Figure 3. Multiple sequence alignment of oat TCP proteins lacking a complete bHLH domain. (A) The TCP proteins of oat are derived from the oat variety 'SFS'; (B) The TCP proteins of oat are derived from the oat variety 'Sang'; (C) The TCP proteins of oat are derived from the oat variety 'OT3098'.

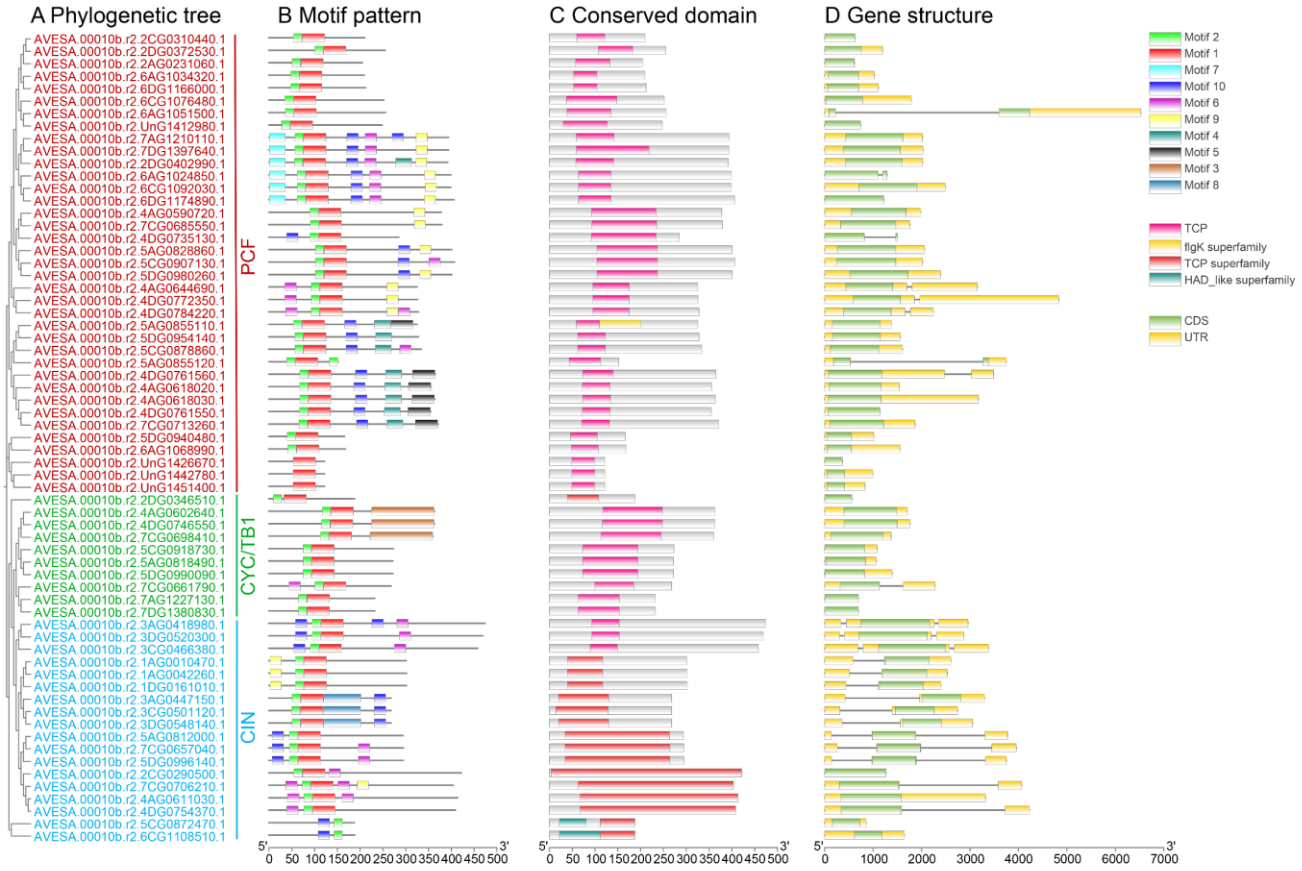

Supplementary Figure 4. Phylogenetic analysis, motif patterns, conserved domains, and gene structure of TCP genes in the 'Sang'. (A) Neighbor-Joining tree of oat TCP proteins; (B) Motif patterns, with motifs numbered 1-10 and represented by different colored boxes; (C) Conserved domains identified in the oat TCP proteins; (D) Gene structure representation, where CDS and UTRs are depicted as green and yellow boxes, respectively, and introns are shown as black lines.

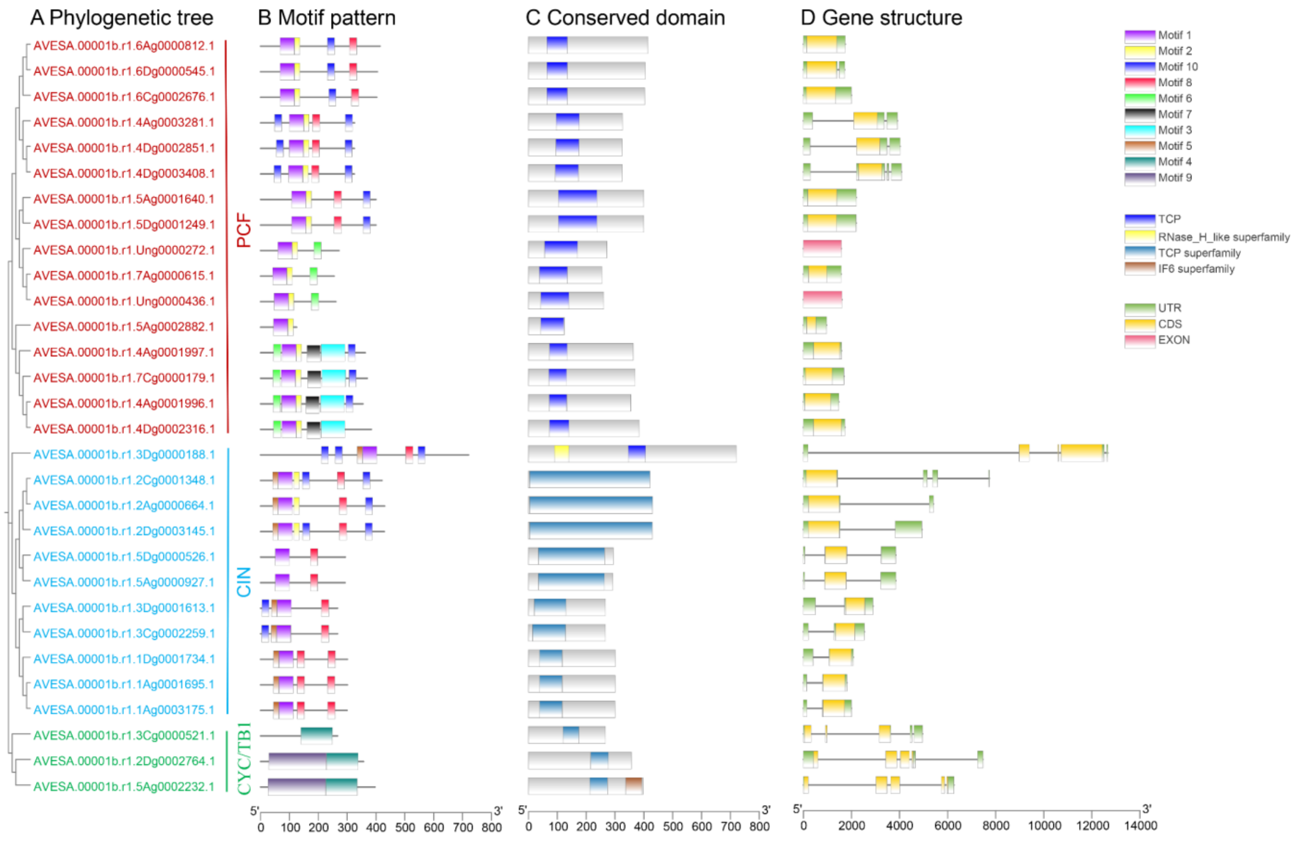

Supplementary Figure 5. Phylogenetic analysis, motif patterns, conserved domains, and gene structure of TCP genes in the 'OT3098'. (A) Neighbor-Joining tree of oat TCP proteins; (B) Motif patterns, with motifs numbered 1-10 and represented by different colored boxes; (C) Conserved domains identified in the oat TCP proteins; (D) Gene structure representation, where UTRs, CDS, and EXONs are depicted as green, yellow, and pink boxes, respectively, and introns are shown as black lines.

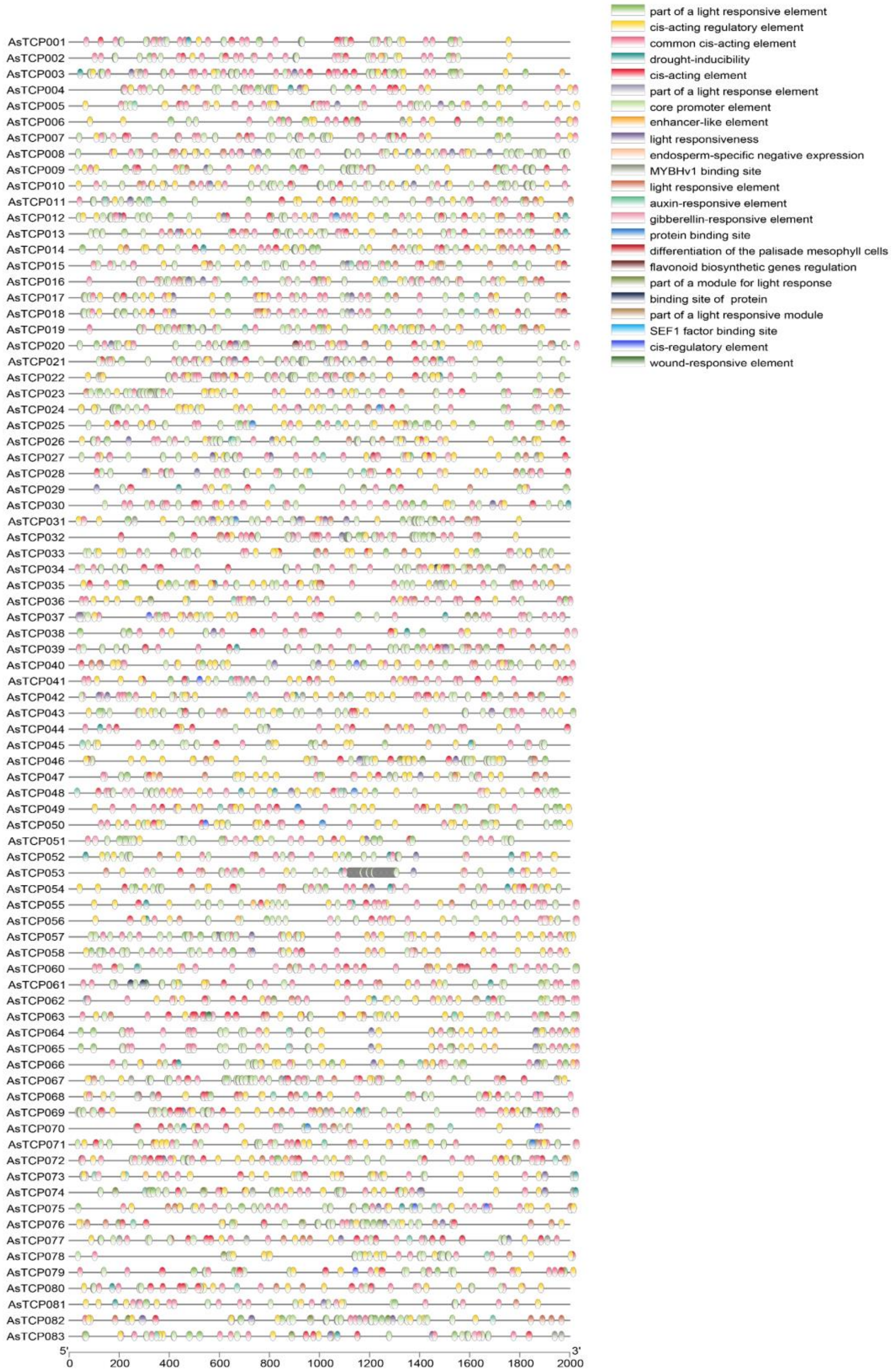

Supplementary Figure 6. Distribution of cis-acting elements in the promoters of *TCP* gene family in the 'SFS'.

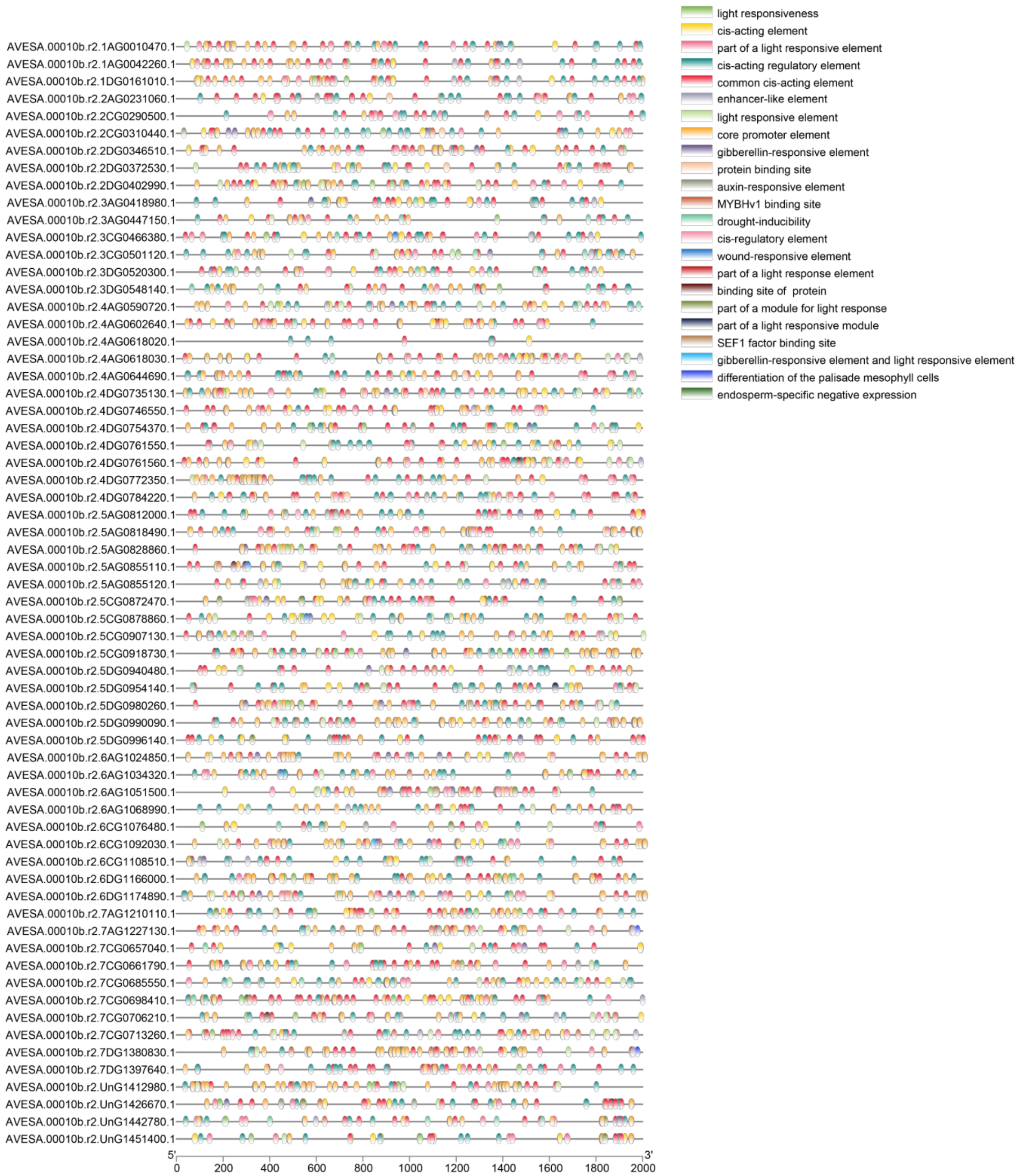

Supplementary Figure 7. Distribution of cis-acting elements in the promoters of *TCP* gene family in the 'Sang'.

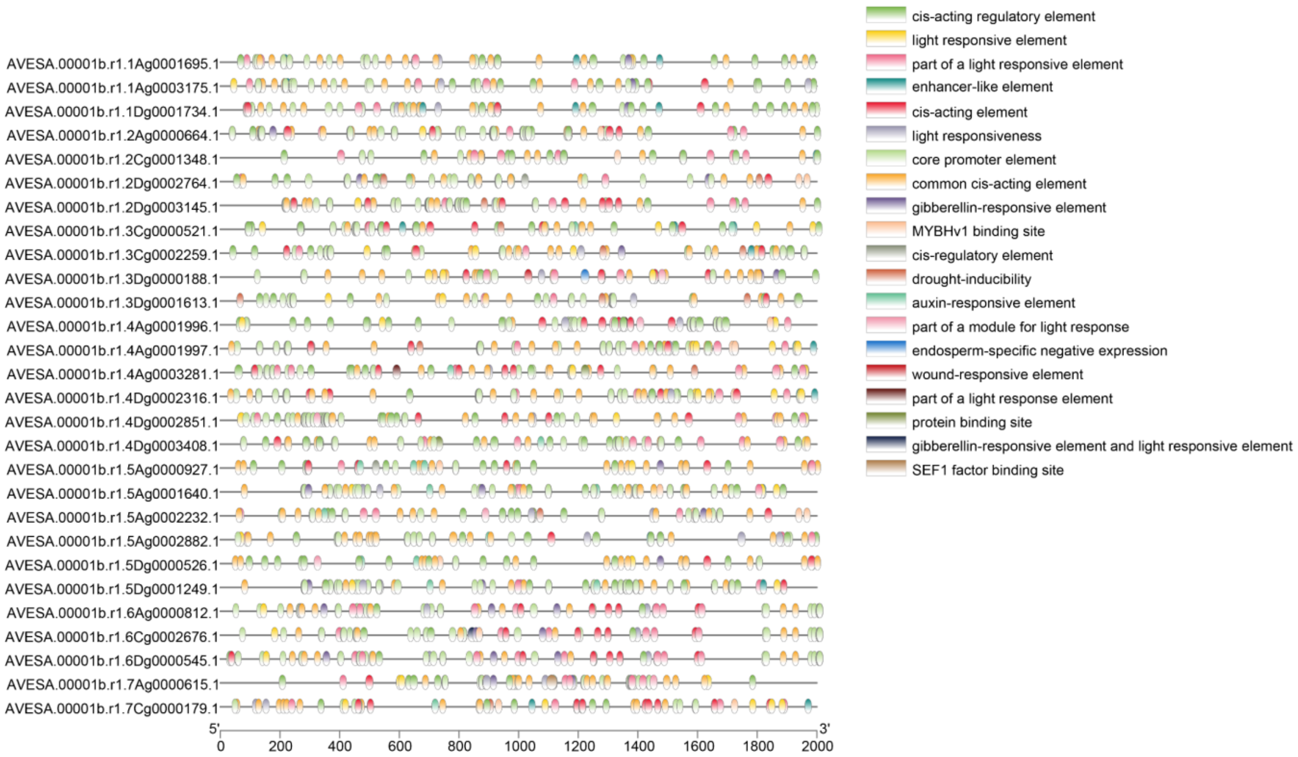

Supplementary Figure 8. Distribution of cis-acting elements in the promoters of *TCP* gene family in the 'OT3098'.

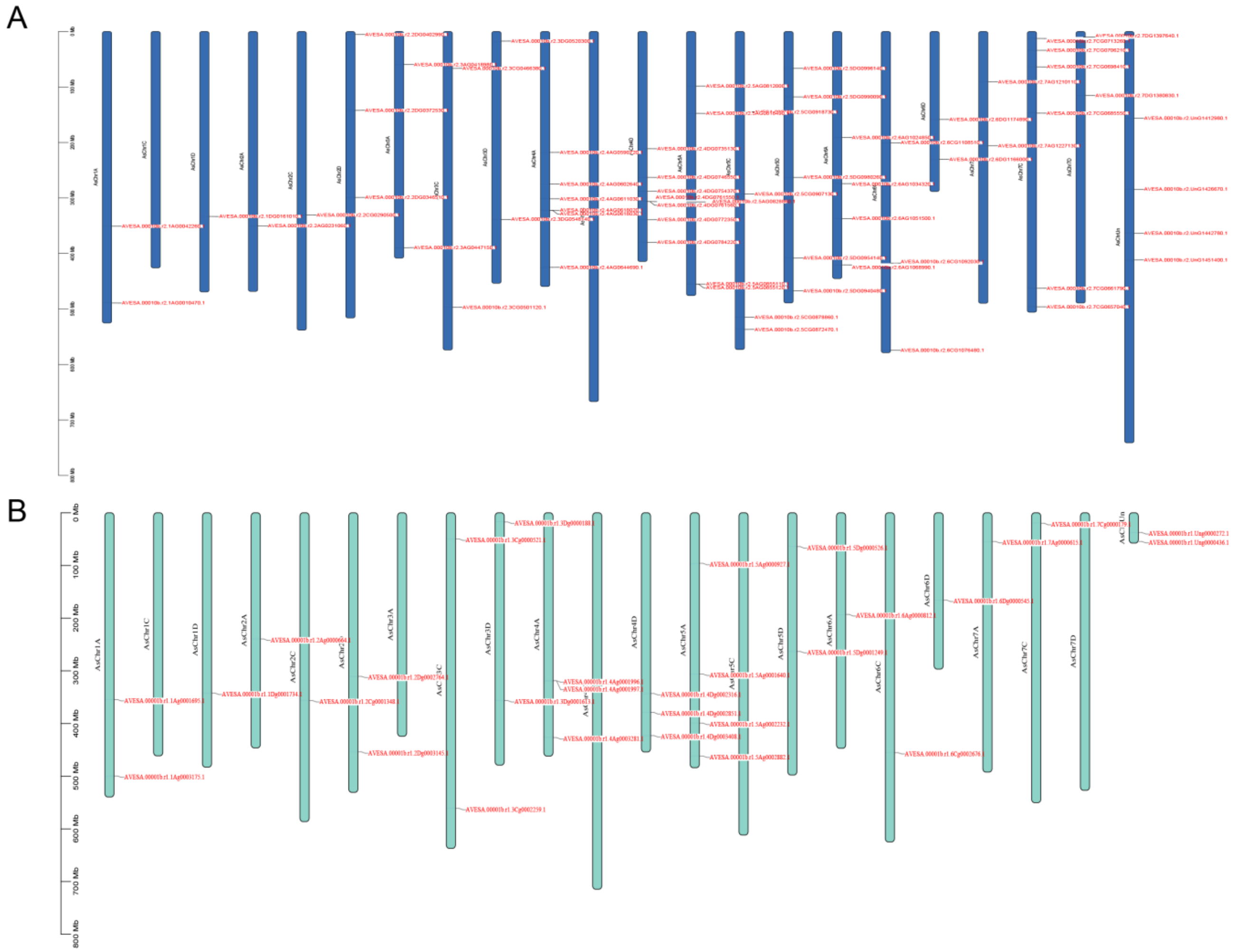

Supplementary Figure 9. Chromosomal localization of *TCP* genes in oats. (A) Distribution of *TCP* genes in oats from the oat variety 'Sang' on chromosomes; (B) Distribution of *TCP* genes in oats from the oat variety 'OT3098' on chromosomes.

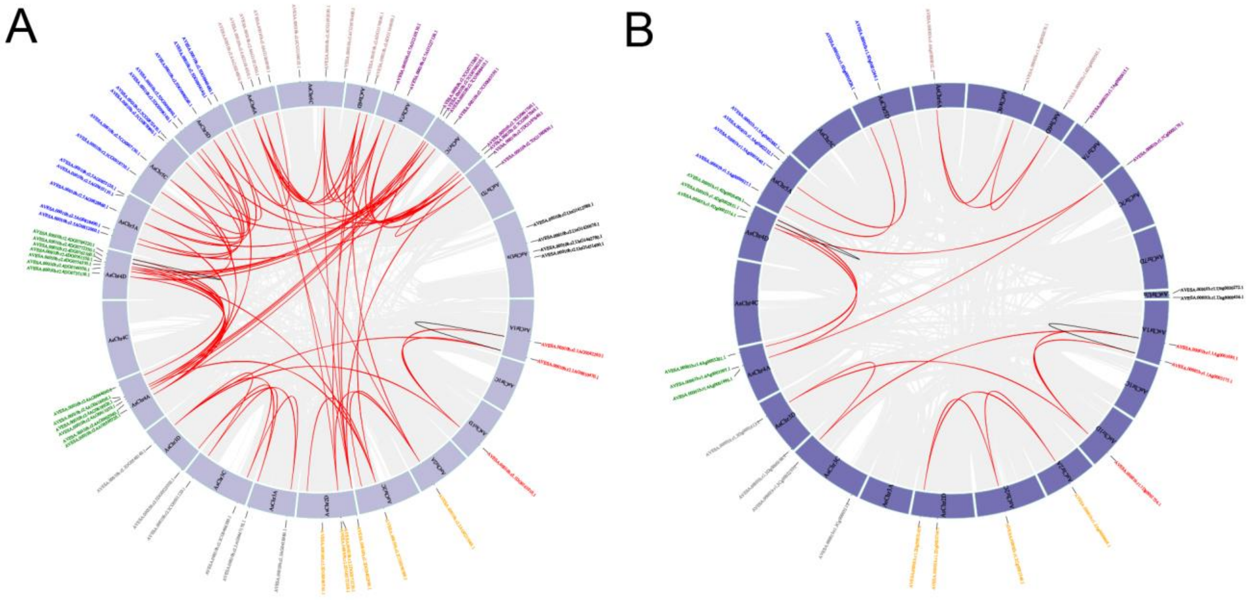

Synteny analysis of of *TCP* genes in oats. Red colored lines indicate segmental duplication gene pairs, black lines indicate tandem duplication pairs. (A) The *TCP* genes of oat are derived from the oat variety 'Sang'; (C) The *TCP* genes of oat are derived from the oat variety 'OT3098'.

1.2 Supplementary Tables

Table S1. The names and sequence information of the primers used in this study.

Table S2. Homology analysis between *AsTCP* genes of 'SFS', 'Sang' and 'OT3098' found in this study.

Table S3. Sequence information for members of the *TCP* gene family in the 'SFS', 'Sang' and 'OT3098'.

Table S4. Identification and analysis of *TCP* gene family members in the 'SFS', 'Sang', and 'OT3098'.

Table S5. Analysis of conserved motifs of TCP protein in the 'SFS'.

Table S6. Cis-acting elements of *AsTCP* genes in the 'SFS'.

Table S7. Segmental and tandem duplications of *TCP* gene pairs in oat.

Table S8. One-to-one homology between *TCP* genes in six plants and oats.

Table S9. *miRNA-AsTCP* prediction.

Table S10. Expression patterns of *AsTCP* genes under silicon-mediated drought stress alleviation.

Table S11. Expression pattern of *AsTCP* genes under salt stress.
